# Supplementary material for: Riboswitch and small RNAs modulate btuB translation initiation in Escherichia coli and trigger distinct mRNA regulatory mechanisms
Source: Nucleic Acids Res. 2024 May 14;52(10):5852–65. doi: 10.1093/nar/gkae347 (PMC11162775; doi:10.1093/nar/gkae347)
Supplement: gkae347_Supplemental_File [file gkae347_supplemental_file.pdf]

Riboswitch and small RNAs modulate *btuB* translation initiation in *Escherichia coli* and trigger distinct mRNA regulatory mechanisms

Laurène Bastet[1#¶], Alexey P. Korepanov[2#&], Jonathan Jagodnik[2], Jonathan P. Grondin[1], Anne-Marie Lamontagne[1], Maude Guillier[2]* and Daniel A. Lafontaine[1]*

[1]Department of Biology, Faculty of Science, Université de Sherbrooke, Sherbrooke, Quebec, Canada, J1K 2R1.

[2]Expression Génétique Microbienne, UMR8261 CNRS, Université Paris Cité, Institut de Biologie Physico-Chimique, 75005 Paris, France.

#These authors contributed equally.

[¶]Present address: Venvirotech Biotechnology SL, 08130 Santa Perpètua de Modoga, Barcelona, Spain.

[&]Present address: Institute of Protein Research RAS, 142290 Pushchino, Moscow Region, Russia

*Corresponding authors. E-mail: maude.guillier@ibpc.fr and daniel.lafontaine@usherbrooke.ca

Keywords: Small RNA, riboswitch, OmrA, AdoCbl, Hfq

Supplementary Information

Strains and plasmids constructions

Strains and plasmids constructed and used in this study are listed in the Supplementary Table S1. Corresponding DNA oligonucleotides (oligos) are summarized in the Supplementary Table S2.

Genetic manipulations for the construction of new gene knockouts and reporter fusions were carried out using a recombineering approach that employs phage λ Red recombination functions as described in detail in (1). Simple allelic exchange was performed using the generalized P1 transduction as described in (2). Strains were cultivated in liquid LB, LB agar plates, BYE (LB without salt) agar plates supplemented with 6% (w/v) sucrose (BS plates) or liquid and solid minimal medium A. Acid LB (pH 4.7) was made by addition of HCl. When needed, media were supplemented with an appropriate antibiotic. Unless otherwise stated, kanamycin (Kan) at 50 µg/mL, chloramphenicol (Cam) at 20 µg/mL, ampicillin (Amp) at 150 µg/mL or tetracycline (Tet) at 10 µg/mL were utilized for plasmid maintenance. For selection of strains carrying a single copy of a drug resistance marker the concentrations were twice as low. During the P1 transduction, selectable plates also contained 5 mM sodium citrate.

Designing an E. coli strain for construction of chromosomal mScarlet fluorescent reporter fusions

The strain OK510 was engineered for constructing mScarlet fusions. It is a DJ624 (MG1655 *ΔlacX74 mal::lacIq*) derivative that carries a *mini-λ-Tet* prophage (3), which provides λ Red recombination functions that are induced at 42°C, and an *mScarlet* locus placed into the 6 bp intergenic region *argG-yhbX*. This locus (see Supplementary Figure S15) comprises a synthetic bidirectional transcriptional terminator L3S2P21 (TT1) (4), an inactive $P_{LtetO-1}$ promoter (5) that is deprived of the -10 region, the Pcat-*cat-sacB* cassette expressed in the opposite direction to that of following 'mScarlet region (mScarlet-I ORF missing the translation initiation codon), and the *nptII* ORF, which is transcriptionally coupled to *'mScarlet* and flanked by two FRT sites. The FRT sites allow for optional

elimination of the *nptII* ORF *via* Flp-FRT recombination. The last element of the locus is a natural bidirectional transcriptional terminator, ECK120026481 (TT2) (4). Hence, the strain features Tet^R (due to the presence of *mini-λ-Tet*), $Sucr^S$, Cam^R and Kan^S as both '*mScarlet* and *nptII* genes are not expressed.

The construction of mScarlet fusions was made with PCR cassette containing ~40 bp homologies on both ends that allow for recombining with its 5'-end within the inactive $P_{LtetO-1}$ restoring the -10 region, and with its 3'-end within the '*mScarlet* (restoring the translation initiation codon), so that it replaces the Pcat-*cat-sacB* sequences. This generates a translational fusion to *mScarlet*, which is expressed from a strong constitutive (in the absence of the Tet repressor) promoter $P_{LtetO-1}$ and provides expression of the downstream *nptII* ORF that renders the recombinants resistant to kanamycin. Thus, the recombinants can be selected for both resistance to sucrose and kanamycin, which greatly reduces occurrence of false-positive clones as compared to selection on sucrose alone. Selection at 37°C also facilitates elimination the *mini-λ-Tet* prophage in the majority of recombinants.

This OK510 strain was made through multiple steps of recombineering, summarized in Supplementary Figure S15. First, a *cat-sacB* cassette was amplified in 3 steps in order to add upstream the inactive $P_{LtetO-1}$ promoter lacking the -10 region, as well as upstream and downstream transcription terminators (TT1 and TT2, respectively) and homology regions to the argG/yhbX locus. The first step PCR template was the genomic DNA of a strain carrying a *cat-sacB* cassette and the primers used are (i) Ptetno-10-cat-for and Ter-catsacRev (1st step), (ii) Ter-Ptet-for and yhbX-Ter-rev (2nd step = reamplification of PCR of 1st step) and (iii) argG-Ter-for and yhbX-Ter-rev (3rd step = reamplification of PCR of 2nd step). This last PCR product was recombined in strain MG1432 and recombinants were selected on LB-Cam at 30°C, and then checked for sucrose sensitivity and Tet resistance (to ensure that they kept the mini-λ Tet for further recombineering). The structure of the locus was checked by sequencing and the resulting strain was called MG2346. In a second recombineering step, the *nptII* gene followed by an FRT site (PCR product amplified from genomic DNA with primers sacB-KanR-For and FRT-sacB-Rev) was introduced downstream of the *sacB* gene, giving rise to the strain MG2348 after selection on Kan and

sequencing of the locus. The third step consisted in replacing the *cat-sacB* cassette by the mScarlet gene devoid of the start codon, and followed by an FRT site. This was again done by recombineering, into strain MG2348 this time, using a PCR product amplified from the pNF02-mScI plasmid (a gift from N. Fraikin and L. Van Melderen, (6)) with primers Ptetno-10-mSC-For and mSc-FRT-Rev, and then reamplified with primers Ptet-55-12For and postFRT-KanR-Rev to increase the length of the homology regions. Recombinants were selected at 30°C on BS plates, and checked for CamS and TetR. Clones whose structure of this TT1-Ptet(no-10)-‘mScarlet(no AUG)-FRT-nptII-FRT-TT2 region was confirmed by sequencing were found to be KanS; out of those, strain MG2352 was used for the subsequent step.

At the last step, the Pcat-*cat-sacB* cassette, made with oligos AK411 and AK412 using chromosomal DNA of NC397 strain (7) as a template, was recombined into MG2352 with subsequent selection of recombinants on LB-Cam plates at 30°C giving rise to the strain OK509. To avoid mutations that could have accumulated upon multiple recombineering passages during construction of the OK509 strain, the ‘*mScarlet* locus of OK509 was transferred into the initial strain, MG1432, selecting transductants on LB-Cam plates at 30°C. The resulting strain, OK510, was used for construction of the mScarlet fusions used in this study.

mScarlet fusions construction

To obtain $P_{LtetO\text{-}1}$-$sdhC_{-222+39}$-$mScarlet_{+4}$, $P_{LtetO\text{-}1}$-$ompR_{-35+717}$-$mScarlet_{+4}$, and $P_{LtetO\text{-}1}$-$btuB_{-240+210}$-$mScarlet_{+4}$, corresponding *loci* of MG1655 chromosome were PCR amplified with following pairs of oligos: Ptet-sdhC-222for and sdhC+39-mScrev (then, re-amplified with Ptet-55-12For and sdhC+39-mScrev); 5'PtetompR-35+30-lacZ and ompR+717-mScrev (then, re-amplified with Ptet-55-12For and ompR+717-mScrev); 5’PtetBtuB-240 and AK451 (then, re-amplified with Ptet-55-12For and AK420). Resulting PCR products were recombined into the OK510 strain. Recombinants were selected at 37°C on BS plates supplemented with 6 μg/mL kanamycin. The recombinant colonies were checked for fluorescence and sensitivity to tetracycline (elimination of the *mini-λ-Tet*). The upstream

mScarlet regions of the obtained OK528, OK529 and OK572 strains were sequenced with oligos AK387 and AK418.

lacZ fusions construction

Construction of *lacZ* fusions was done by replacing a *cat-sacB* cassette upstream of *lacZ* by recombineering into strain PM1205 ((8), for construction of P_{BAD} driven fusions) or MG1508 ((9), for construction of $P_{LtetO-1}$ driven fusions) and OK868 (an MG1508 variant with the Δ*lacY*::FRT deletion). At low IPTG concentrations, its intracellular levels are largely dependent on the LacY-mediated uptake. As *lacZ* and *lacY* are co-expressed, the LacY levels (and IPTG uptake activity) vary according to the *lacZ* fusion activity. To uncouple the intracellular IPTG levels from the activity of a *lacZ* fusion, *i.e.* to provide equal induction conditions in different *lacZ* fusion strains, Δ*lacY*::FRT background was used (for Supplementary Figure S12). To this end, the Δ*lacY*::FRT-*nptII*-FRT allele was PCR amplified from JW0334 chromosome using AK51 and AK52 primers, and the resulting PCR product was recombined into MG1508, selecting on Kan (generating OK865). The *nptII* was eliminated from OK865 via Flp-FRT recombination (leaving Δ*lacY*::FRT scar) using pCP20 plasmid giving rise to OK866. Then, the $P_{LtetO-1}$-*cat-sacB-lacZ*-Δ*lacY*::FRT region was transferred into MG1432 via P1 transduction selecting on Cam (at 30°C, to preserve the *mini-λ-Tet*), and the obtained Δ*lacY*::FRT version of MG1508 was referred to as OK868.

In general, the PCR products were obtained using MG1655 genomic DNA as a template, and, when needed, the length of the homology regions was extended by re-amplification with primers Ptet-55-12For or lacZ28-66rev as necessary. The mutations EP1, EP2, EP3, M9, M11 and mutH1 were present on the primers, and the same was true for the low$P_{LtetO-1}$ mutant that changes the -35 region consensus of $P_{LtetO-1}$ from TTGACA into TGGACA. For the construction of the P_{BAD}-*btuB-lacZ* transcriptional fusions, successive PCR reactions introduced, downstream of the selected *btuB* region, an in-frame sequence encoding a DPAF peptide terminated by a stop codon, followed by *lacZ* sequence starting 17 nts before *lacZ* translation initiation codon.

To produce Δ*lacY*::FRT versions of the *lacZ* fusions, the fusion regions were PCR amplified with primers Ptet-55-12For and lacZ28-66rev using corresponding WT *lacY* fusion strains chromosome as a template. The PCR products were recombineered into OK868.

$P_{LtetO-1}$-driven versions of the transcriptional fusions were obtained by recombineering in MG1508. In each case, a relevant PCR product for recombineering was made in two steps: 1) amplification of the fusion region with primers 5'PtetBtuB-240 and lacZ28-66rev using a corresponding P_{BAD}-driven fusion strain chromosome as a template, and 2) re-amplification of the step 1 PCR product with Ptet-55-12For and lacZ28-66rev.

Recombinants were selected on BS plates supplemented with 0.002% X-gal at 37°C, picking blue colonies and checking them for sensitivity to chloramphenicol (loss of the *cat-sacB* cassette) and tetracycline (loss of the *mini-λ-Tet*). The *lac* loci of resulting strains were verified by sequencing.

Constructing rne131 derivatives of transcriptional $P_{LtetO-1}$-btuB-lacZ fusion strains

The *rne131* allele is commonly co-transduced with the *zhe-726*::Tn*10* which allows selection of tetracycline resistant transductants, which would contain the *rne131* allele at approximately 50% frequency. Unfortunately, the $P_{LtetO-1}$ promoter was designed to be repressed by the TetR repressor of Tn*10*. To provide constitutive expression of the $P_{LtetO-1}$-*btuB-lacZ* fusions in the *rne131* context, we first transferred the *rne131/zce726*::Tn*10* from the HfrG6Δ*lac*12RevES191 *rne131 zce-726*::Tn*10* strain into DJ624 generating OK837. Then, the *zce726*::Tn*10* was replaced by the Δ*ptsG*::FRT-*nptII*-FRT of JW1087 via P1 transduction giving rise to OK838. The *rne131*/Δ*ptsG*::FRT-*nptII*-FRT region was then transferred into the $P_{LtetO-1}$-*btuB-lacZ* fusion strains selecting on Kan. As *rne131* is also co-transduced with ~50% frequency, both *rne131* and WT *rne* transductants were obtained (see OK855-OK860), which were used for the experiments in Supplementary Figure S4C. Thus both, the WT and the *rne131* strains there carried the Δ*ptsG*::FRT-*nptII*-FRT allele.

Constructing omrA and omrB and omrAB deletion strains

PCR cassettes for generating *ΔomrA*::*nptI* and *ΔomrB*::*nptI* were obtained using pairs of primers, OmrA-kan5 + OmrA-Kan3 and OmrB-kan5 + OmrB-kan3, respectively. As a template, chromosomal DNA of a strain carrying an *nptI* gene was used. The cassettes were recombined into NM300 strain selecting recombinants on LB-Kan at 37°C. Loss of mini-λ-Tet was verified by sensitivity to Tet. The deletions in resulting strains MG1001 (*ΔomrA*::*nptI*) and MG1002 (*ΔomrB*::*nptI*) were verified by PCR and sequenced with oligos seqOmrBfor and seqOmrArev. The obtained *ΔomrA*::*nptI* and *ΔomrB*::*nptI* and the previously published double deletion *ΔomrAB*::*nptI* of MG1003 (10) were transferred to JJ416, generating OK615, OK616 and JJ426, respectively. Same deletions were transferred to JJ425 giving rise to OK617, OK618, and JJ427, respectively.

Elimination of the nptII ORF from FRT-nptII-FRT cassettes

Unnecessary *nptII* ORFs originating from the OK510 derivatives and from Keio collection knockouts (11) used in this study were eliminated *via* Flp-FRT recombination. To this end, cells were transformed with the pCP20 plasmid (12). The plasmid provides *in trans* synthesis of flippase (Flp) and possesses a thermo-sensitive replication origin. Transformants were selected at 30°C on LB-Cam (10 µg/mL) plates supplemented with chloramphenicol and purified once on the same medium. Then, an individual transformant colony was grown overnight in 10 mL of LB medium at 42°C and plated on LB at ~10^2 cfu per plate 37°C. After such passage vast majority of clones were both KanS and CamS.

Constructing Hfq variant strains carrying the mScarlet fusions under study

The construction of strains carrying different *hfq* mutations is based on (13), with minor modifications to combine these different alleles with mScarlet fusions. First, the *ΔargG*::FRT-*nptII*-FRT allele was transferred from JW3140 (from the Keio collection (11)) to DJ624 by P1 transduction to generate strain OK523. This OK523 strain was then cured of *nptII* as described above generating OK530. The *Δhfq*::*cat-sacB* allele was co-transduced with nearby *purA*::FRT-*nptII*-FRT from DJS2604 (from D. Schu, NIH) to OK530 selecting on LB-Cam, thus giving rise to OK564. The *hfq* alleles were transferred

from DJS2609 (*hfq* WT), DJS2927 (*Δhfq*), KK2560 (*hfqQ8A*), KK2561 (*hfqR16A*), and KK2562 (*hfqY25D*) to OK564 selecting transductants on minimal medium A-agar plates (16) supplemented with 0.2% (w/v) of glucose and 20 µg/mL of L-arginine (the acceptor strain, OK564, is a purine and arginine auxotroph). Transfer of the *hfq* alleles was verified by PCR with *hfq* check oligos mHfqout and antiHfqout, or with the mHfqout and one of the *hfq* point mutant-specific oligos, AK430 (Q8A-specific), AK431 (R16A-specific), and AK432 (Y25D-specific). The resulting strains, OK581 (*hfq* WT), OK582 (*Δhfq*), OK583 (*hfqQ8A*), OK584 (*hfqR16A*), and OK585 (*hfqY25D*), served as acceptors for transferring the mScarlet fusion variants. To do so, the fusion strains OK528, OK529 and OK572 were cured of the *nptII* cassette, thus generating strains OK560, OK561 and OK577, respectively. Then, each fusion was transduced into the five *hfq* variant strains. Arginine prototroph transductants were selected at 37°C on minimal A-agar plates supplemented with 0.2% glucose (mScarlet locus is 100% co-transduced with the wild type *argG* allele). The resulting strains, OK586 to OK605, are listed in the Supplementary Table S1.

Cloning Hfq under an IPTG-inducible promoter

A pCAS18 plasmid was given by M. Springer. It carries *E. coli rpsR* gene that was cloned in a pCA24N (14) using *BseR*I and HindIII restriction sites (*Sal*I site is present between the *rps*R stop codon and *Hind*III site). A unique *Nde*I restriction site was made at the *rpsR* start codon in pCAS18 by PCR with primers AK74 and AK75 giving rise to pCAS18n. Then, a 233 bp *Nde*I-*Sal*I region of pCAS18n containing *rpsR* was replaced by a 354 bp Hfq-containing fragment of the pHfq (15). The resulting plasmid that provides IPTG-induced expression of Hfq was referred to as pNK139. Thus, the pNK139 allows IPTG-inducible synthesis of Hfq from the $P_{T5\text{-}lac}$ promoter, using host RNA polymerase. This provides an opportunity to observe the dose-dependent effect of Hfq on cellular processes such as model fusion expression.

Fluorescence measurements to assess the expression of mScarlet fusions

The *hfq* variant strains OK586-OK590 ($P_{LtetO\text{-}1}$-$btuB_{-240+210}$-$mScarlet_{+4}$), and OK601-OK605 ($P_{LtetO\text{-}1}$-$ompR_{-35+717}$-$mScarlet_{+4}$) were transformed with the pBRplac vector control, pOmrA and pOmrB. The $P_{LtetO\text{-}1}$-$sdhC_{-222+39}$-$mScarlet_{+4}$ *hfq* variant strains (OK596-

OK600) were transformed with pBRplac and pSpot42. In each case transformants were selected on LB-Tet plates at 37°C. DJ624 transformed with pBRplac was utilized as a no-fusion background. After a single purification on the LB-Tet plate, individual transformant colony was inoculated with 400 µL of CAG medium (minimal A salts (16), 0.5% (w/v) glycerol, 0.25% (w/v) casamino acids, 1 mM $MgSO_4$) supplemented with Tet and incubated overnight with shaking at 37°C. Next day, 0.4 µL of saturated culture used to inoculate 200 µL of CAG medium supplemented with Tet and 0.25 mM IPTG in the black 96 well µCLEAR F-bottom plate (Greiner, 655090) covered with 50 µl of mineral oil (Sigma, M8410).

Test was run in the CLARIOStar^{PLUS} plate reader (BMG Labtech) at 37°C and 500 RPM. Absorbance at 600 nm and fluorescence (excitation at 560±15 nm, emission at 600±15 nm with 580 nm dichroic filter) was measured every 12 minutes for 16 h. Each experiment was made in triplicate (starting from three independent transformant colonies). Experimental curves expressed as fluorescence normalized to absorbance at 600 nm (normalized fluorescence) versus absorbance at 600 nm are shown in the Supplementary Figures S5 and S7-S11. To generate the histograms, the mean normalized fluorescence and standard deviations were calculated for the three (one per triplicate) time points closest to apparent A_{600}=0.3. Mean normalized fluorescence of the no-fusion control was calculated accordingly and subtracted from the experimental data.

References

1. Sawitzke,J.A., Thomason,L.C., Costantino,N., Bubunenko,M., Datta,S. and Court,D.L. (2007) Recombineering: in vivo genetic engineering in E. coli, S. enterica, and beyond. *Methods in enzymology*, **421**, 171–99.

2. Thomason,L.C., Costantino,N. and Court,D.L. (2007) E. coli genome manipulation by P1 transduction. *Current protocols in molecular biology*, **1**, 1.17.1-1.17.8.

3. Court,D.L., Swaminathan,S., Yu,D., Wilson,H., Baker,T., Bubunenko,M., Sawitzke,J. and Sharan,S.K. (2003) Mini-lambda: a tractable system for chromosome and BAC engineering. *Gene*, **315**, 63–9.

4. Chen,Y.-J., Liu,P., Nielsen,A.A.K., Brophy,J.A.N., Clancy,K., Peterson,T. and Voigt,C.A. (2013) Characterization of 582 natural and synthetic terminators and quantification of their design constraints. *Nature methods*, **10**, 659–64.

5. Lutz,R. and Bujard,H. (1997) Independent and tight regulation of transcriptional units in Escherichia coli via the LacR/O, the TetR/O and AraC/I1-I2 regulatory elements. *Nucleic acids research*, **25**, 1203–10.

6. Fraikin,N., Rousseau,C.J., Goeders,N. and Van Melderen,L. (2019) Reassessing the Role of the Type II MqsRA Toxin-Antitoxin System in Stress Response and Biofilm Formation: mqsA Is Transcriptionally Uncoupled from mqsR. *mBio*, **10**, e0267819.

7. Svenningsen,S.L., Costantino,N., Court,D.L. and Adhya,S. (2005) On the role of Cro in lambda prophage induction. *Proceedings of the National Academy of Sciences of the United States of America*, **102**, 4465–9.

8. Mandin,P. and Gottesman,S. (2009) A genetic approach for finding small RNAs regulators of genes of interest identifies RybC as regulating the DpiA/DpiB two-component system. *Mol Microbiol*, **72**, 551–565.

9. Coornaert,A., Chiaruttini,C., Springer,M. and Guillier,M. (2013) Post-transcriptional control of the Escherichia coli PhoQ-PhoP two-component system by multiple sRNAs involves a novel pairing region of GcvB. *PLoS genetics*, **9**, e1003156.

10. Guillier,M. and Gottesman,S. (2006) Remodelling of the Escherichia coli outer membrane by two small regulatory RNAs. *Mol Microbiol*, **59**, 231–247.

11. Baba,T., Ara,T., Hasegawa,M., Takai,Y., Okumura,Y., Baba,M., Datsenko,K.A., Tomita,M., Wanner,B.L. and Mori,H. (2006) Construction of Escherichia coli K-12 in-frame, single-gene knockout mutants: the Keio collection. *Molecular systems biology*, **2**, 2006.0008.

12. Cherepanov,P.P. and Wackernagel,W. (1995) Gene disruption in Escherichia coli: TcR and KmR cassettes with the option of Flp-catalyzed excision of the antibiotic-resistance determinant. *Gene*, **158**, 9–14.

13. Zhang,A., Schu,D.J., Tjaden,B.C., Storz,G. and Gottesman,S. (2013) Mutations in interaction surfaces differentially impact E. coli Hfq association with small RNAs and their mRNA targets. *Journal of molecular biology*, **425**, 3678–97.

14. Kitagawa,M., Ara,T., Arifuzzaman,M., Ioka-Nakamichi,T., Inamoto,E., Toyonaga,H. and Mori,H. (2005) Complete set of ORF clones of Escherichia coli ASKA library (a complete set of E. coli K-12 ORF archive): unique resources for biological research. *DNA Res*, **12**, 291–299.

15. Vassilieva,I.M., Rouzanov,M.V., Zelinskaya,N.V., Moll,I., Bläsi,U. and Garber,M.B. (2002) Cloning, purification, and crystallization of a bacterial gene expression regulator--Hfq protein from Escherichia coli. *Biochemistry Mosc.*, **67**, 1293–1297.

16. Miller,J.H. (1972) Experiments in Molecular Genetics Laboratory,C.S.H. (ed) Cold Spring Harbor Laboratory Press, NY, USA.

17. Madeira,F., Park,Y.M., Lee,J., Buso,N., Gur,T., Madhusoodanan,N., Basutkar,P., Tivey,A.R.N., Potter,S.C., Finn,R.D., *et al.* (2019) The EMBL-EBI search and sequence analysis tools APIs in 2019. *Nucleic acids research*, **47**, W636–W641.

18. Zuker,M. (2003) Mfold web server for nucleic acid folding and hybridization prediction. *Nucleic Acids Res*, **31**, 3406–3415.

19. Masse,E. and Gottesman,S. (2002) A small RNA regulates the expression of genes involved in iron metabolism in Escherichia coli. *Proc Natl Acad Sci U S A*, **99**, 4620–4625.

20. Masse,E., Escorcia,F.E. and Gottesman,S. (2003) Coupled degradation of a small regulatory RNA and its mRNA targets in Escherichia coli. *Genes Dev*, **17**, 2374–2383.

21. Bastet,L., Chauvier,A., Singh,N., Lussier,A., Lamontagne,A.-M.M., Prévost,K., Massé,E., Wade,J.T. and Lafontaine,D.A. (2017) Translational control and Rho-dependent transcription termination are intimately linked in riboswitch regulation. *Nucleic acids research*, **45**, 7474–7486.

22. Jagodnik,J., Chiaruttini,C. and Guillier,M. (2017) Stem-Loop Structures within mRNA Coding Sequences Activate Translation Initiation and Mediate Control by Small Regulatory RNAs. *Molecular cell*, **68**, 158-170.e3.

23. Ghosh,T., Jahangirnejad,S., Chauvier,A., Stringer,A.M., Korepanov,A.P., Cote,J.-P., Wade,J.T. and Lafontaine,D.A. (2024) Direct and Indirect Control of Rho-Dependent Transcription Termination by the Escherichia coli lysC Riboswitch. *RNA*, 10.1261/rna.079779.123.

24. Majdalani,N., Cunning,C., Sledjeski,D., Elliott,T. and Gottesman,S. (1998) DsrA RNA regulates translation of RpoS message by an anti-antisense mechanism, independent of its action as an antisilencer of transcription. *Proc Natl Acad Sci U S A*, **95**, 12462–12467.

25. Mandin,P. and Gottesman,S. (2010) Integrating anaerobic/aerobic sensing and the general stress response through the ArcZ small RNA. *The EMBO journal*, **29**, 3094–107.

26. Lerner,C.G. and Inouye,M. (1990) Low copy number plasmids for regulated low-level expression of cloned genes in Escherichia coli with blue/white insert screening capability. *Nucleic acids research*, **18**, 4631.

27. Ziolkowska,K., Derreumaux,P., Folichon,M., Pellegrini,O., Régnier,P., Boni,I.V. and Hajnsdorf,E. (2006) Hfq variant with altered RNA binding functions. *Nucleic acids research*, **34**, 709–20.

28. Bindels,D.S., Haarbosch,L., van Weeren,L., Postma,M., Wiese,K.E., Mastop,M., Aumonier,S., Gotthard,G., Royant,A., Hink,M.A., *et al.* (2017) mScarlet: a bright

monomeric red fluorescent protein for cellular imaging. *Nature methods*, **14**, 53–56.

A

```
OmrA CCCAGAGGUAUUGAUUGGUGAGAUUAU--UCGGUACGCUCUUCGUACCCUGUCUCUUGCA 58
OmrB CCCAGAGGUAUUGAUAGGUGAAGUCAACUUCGGGU-----U---GAGCACAUGAAUUACA 52
     *************** *****  * *    ****       *     * *   *   ** **

OmrA CCAACCUGCGCGGAUGCGCAGGUUUUUUUU 88
OmrB CCAGCCUGCGCAGAUGCGCAGGUUUUUUUU 82
     *** ******* ******************
```

B

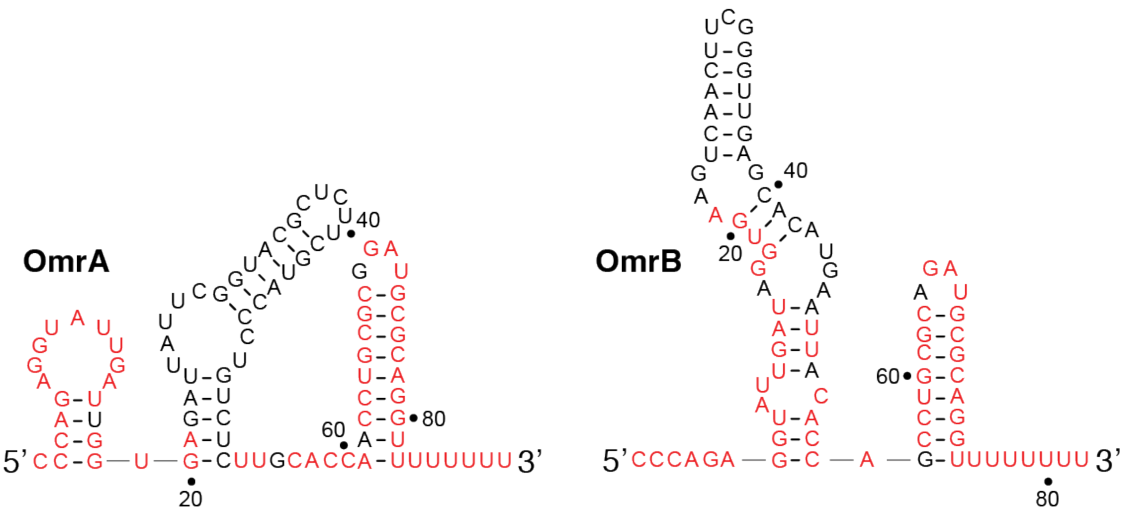

Supplementary Figure S1. Sequences and secondary structures of OmrA and OmrB. **(A)** Sequence alignment of *E. coli* OmrA and OmrB sRNAs performed using Clustal (17). Nucleotides in red represent the 5' and 3' conserved regions of the sRNAs. **(B)** Secondary structures of OmrA and OmrB. The structures have been predicted using Mfold (18). Nucleotides in red indicate the 5' and 3' conserved regions of both sRNAs.

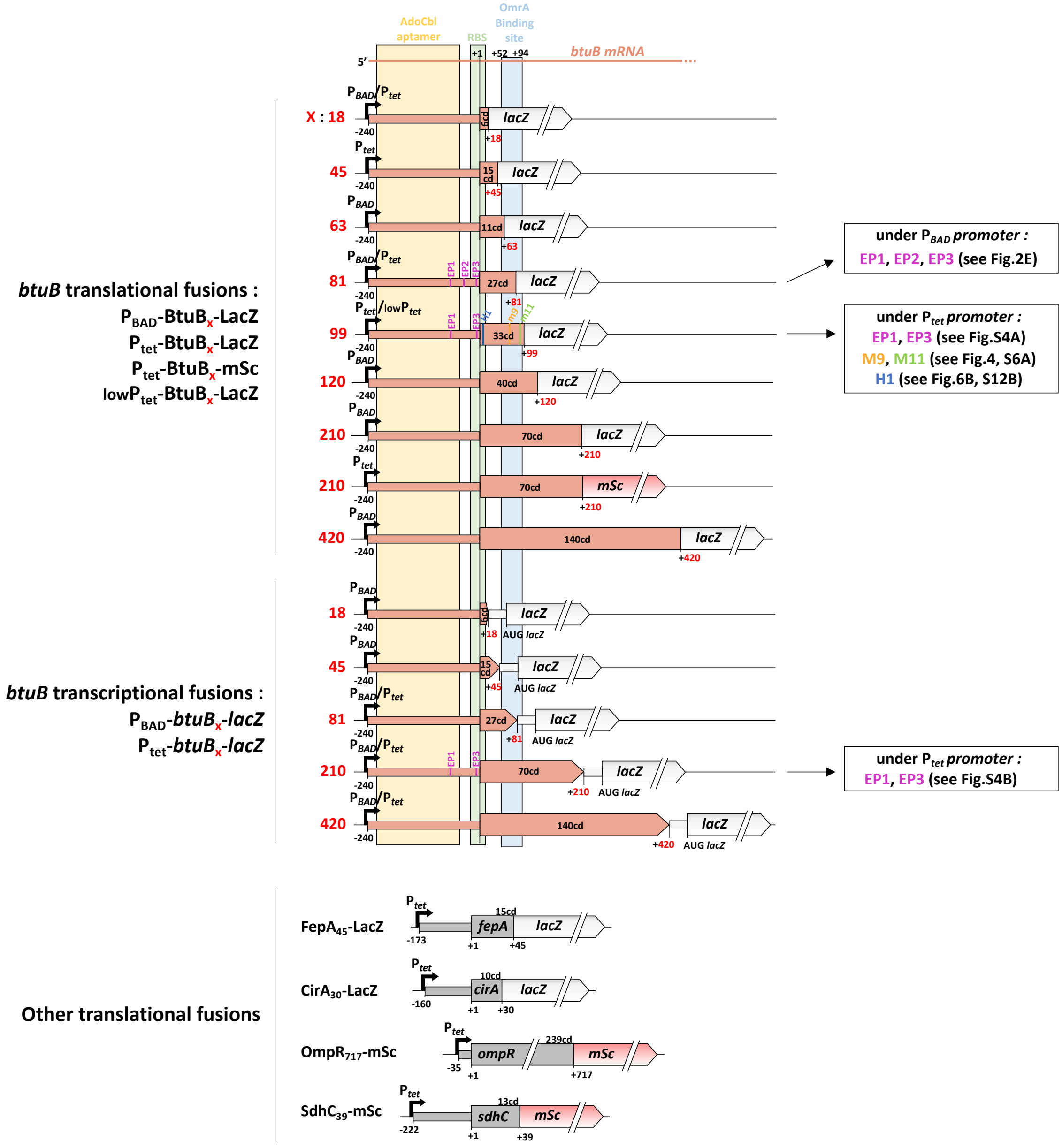

Supplementary Figure S2. Schematics of reporter fusions used in this study. The promoter, the riboswitch (AdoCbl aptamer), the ribosome binding site (RBS), the *btuB* and *lacZ* coding regions are shown. The *btuB* coding region (in nucleotides and codons) is indicated for each construct. The location of the OmrA binding site within the *btuB* coding region determined in this study is shown in blue. Arabinose-inducible (P_{BAD}) or

constitutive $P_{LtetO-1}$ (P_{tet}) promoter is indicated. Transcriptional fusions contain a stop codon following the *btuB* coding region and an additional Shine-Dalgarno (SD) and AUG start codon for *lacZ* expression.

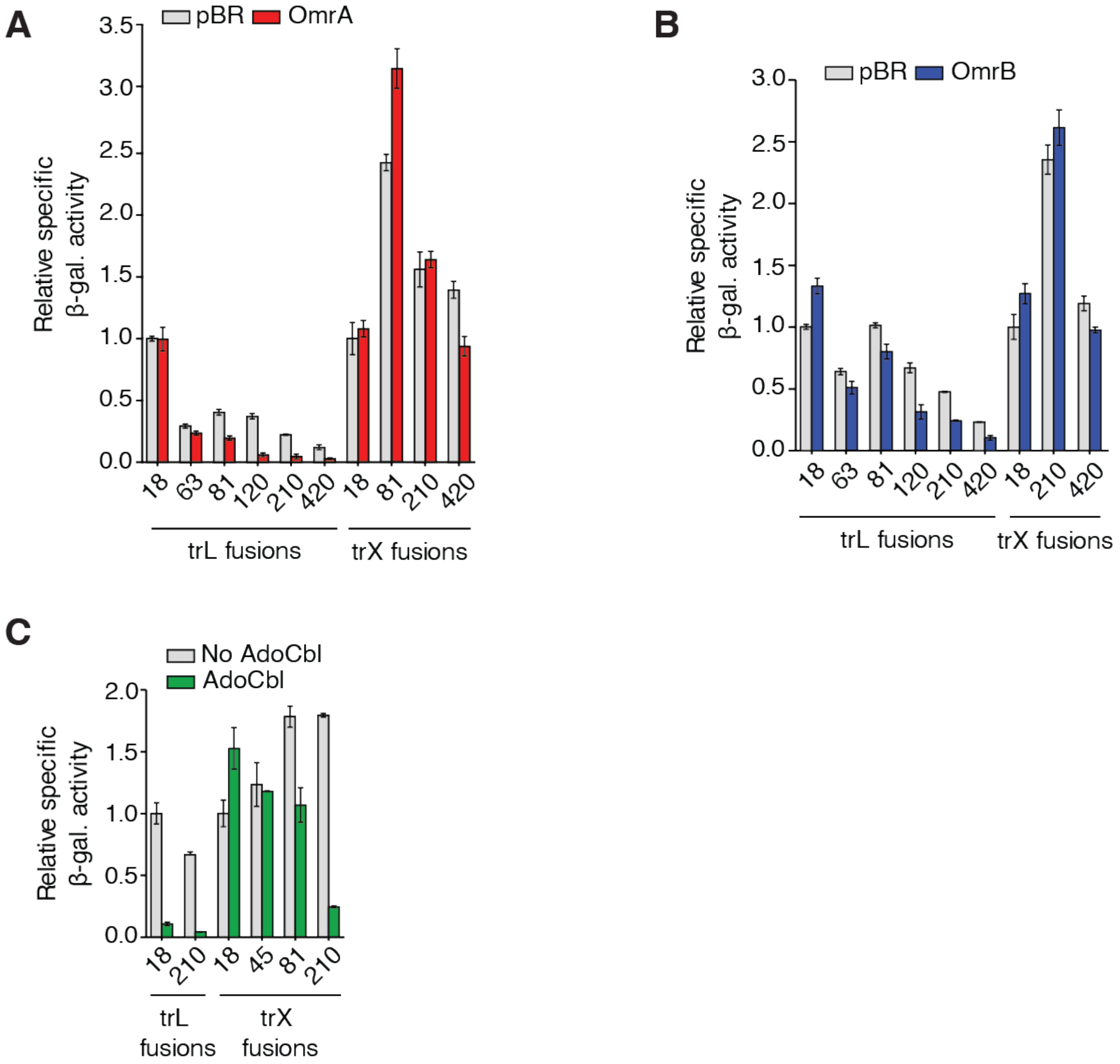

Supplementary Figure S3. sRNA and riboswitch regulation of *btuB* expression. (A-C) β-galactosidase assays of translational BtuB-LacZ (trL) and transcriptional *btuB-lacZ* (trX) fusions with OmrA **(A)**, OmrB **(B)** or AdoCbl **(C)**. The number of nucleotides of *btuB* ORF is indicated for each construct. In each set (trL or trX), the expression of a construct is normalized to the expression of the corresponding 18 nt fusion. This figure represents the normalized data that are in Figures 2B, 2C and 2D.

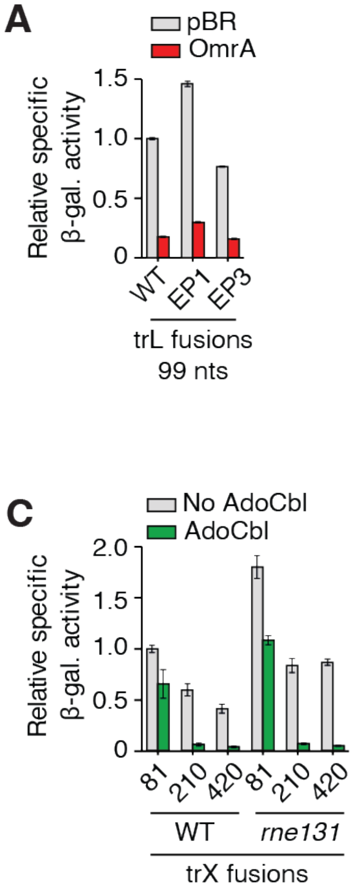

Supplementary Figure S4. Characterization of the molecular mechanism of riboswitch-dependent regulation of *btuB* expression. (A) β-galactosidase assays of translational EP1 and EP3 BtuB-LacZ (trL) fusions with and without OmrA. **(B)** β-galactosidase assays of transcriptional EP1 and EP3 *btuB-lacZ* (trX) fusions with and without AdoCbl. **(C)** β-galactosidase assays of transcriptional *btuB-lacZ* (trX) fusions with and without AdoCbl in WT and *rne131* mutant strain. The number of nucleotides of *btuB* ORF is indicated for each construct. **(D)** β-galactosidase assays of translational WT and M9 BtuB-LacZ (trL) fusions with and without AdoCbl.

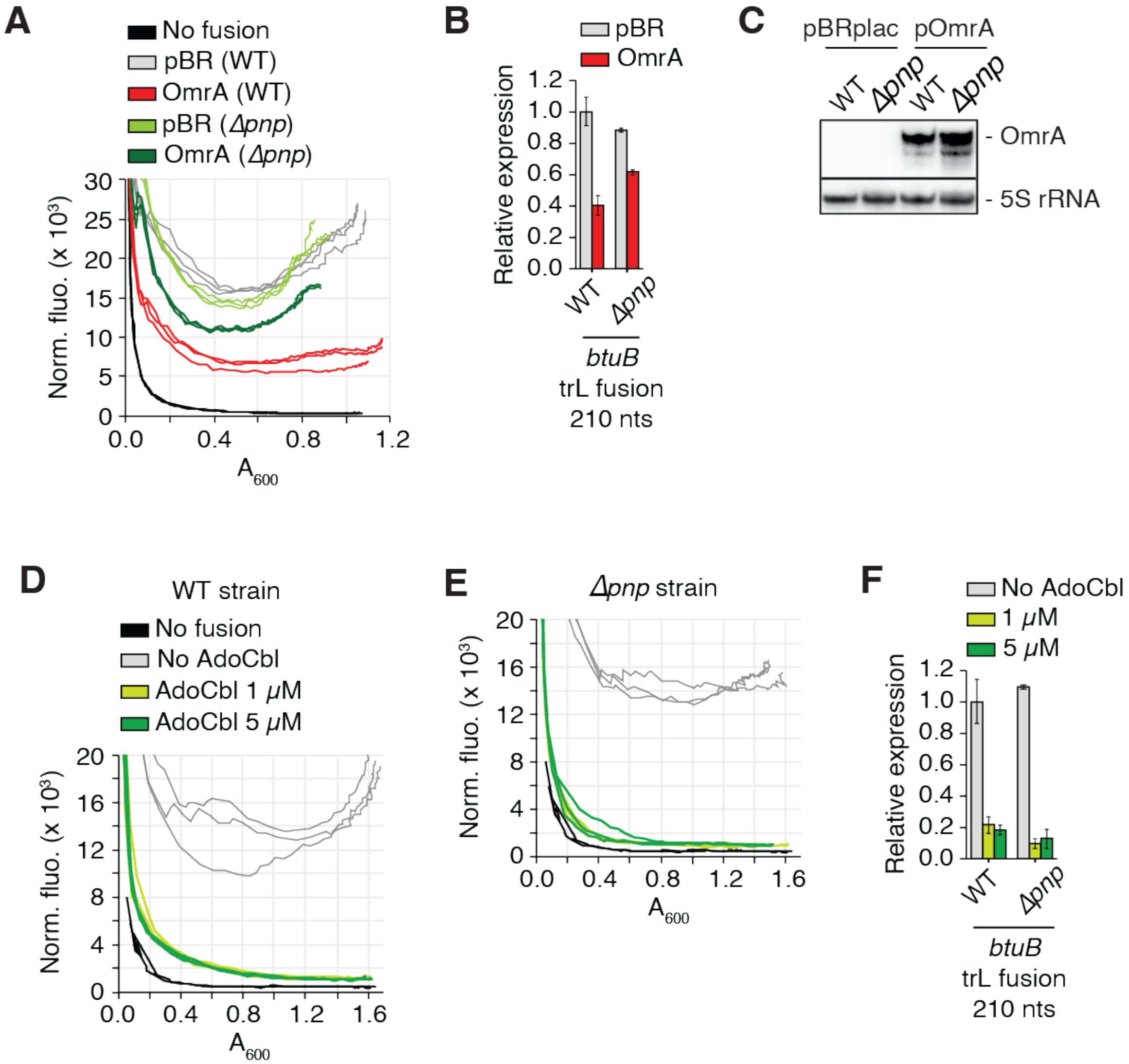

Supplementary Figure S5. The control of *btuB* by OmrA is impaired in the absence of PNPase. (A) The fluorescence of the BtuB210-mScarlet translational fusion was followed in WT and *pnp* deleted cells, transformed with a vector control (pBR) or an OmrA overproducing plasmid. The fluorescence of a strain that does not express the mScarlet gene was followed as a control for the fluorescence background. The curves show normalized fluorescence plotted against the absorbance at 600 nm for all samples (in triplicate). **(B)** The average value and standard deviation of normalized fluorescence at an absorbance of 600 nm closest to 0.3 is shown on the histograms. **(C)** The levels of OmrA

in WT or *pnp* deleted cells were analyzed by Northern-blot in an independent experiment. **(D, E, F)** The fluorescence of the $BtuB_{210}$-mScarlet fusion was measured in WT **(D)** or *pnp* deleted cells **(E)** grown in the absence or in the presence of AdoCbl at a final concentration of 1 or 5 μM. Graphic representation is as in panel **A**, and data from panels **D** and **E** are processed and summarized on panel **F** the same way as for panel **B**.

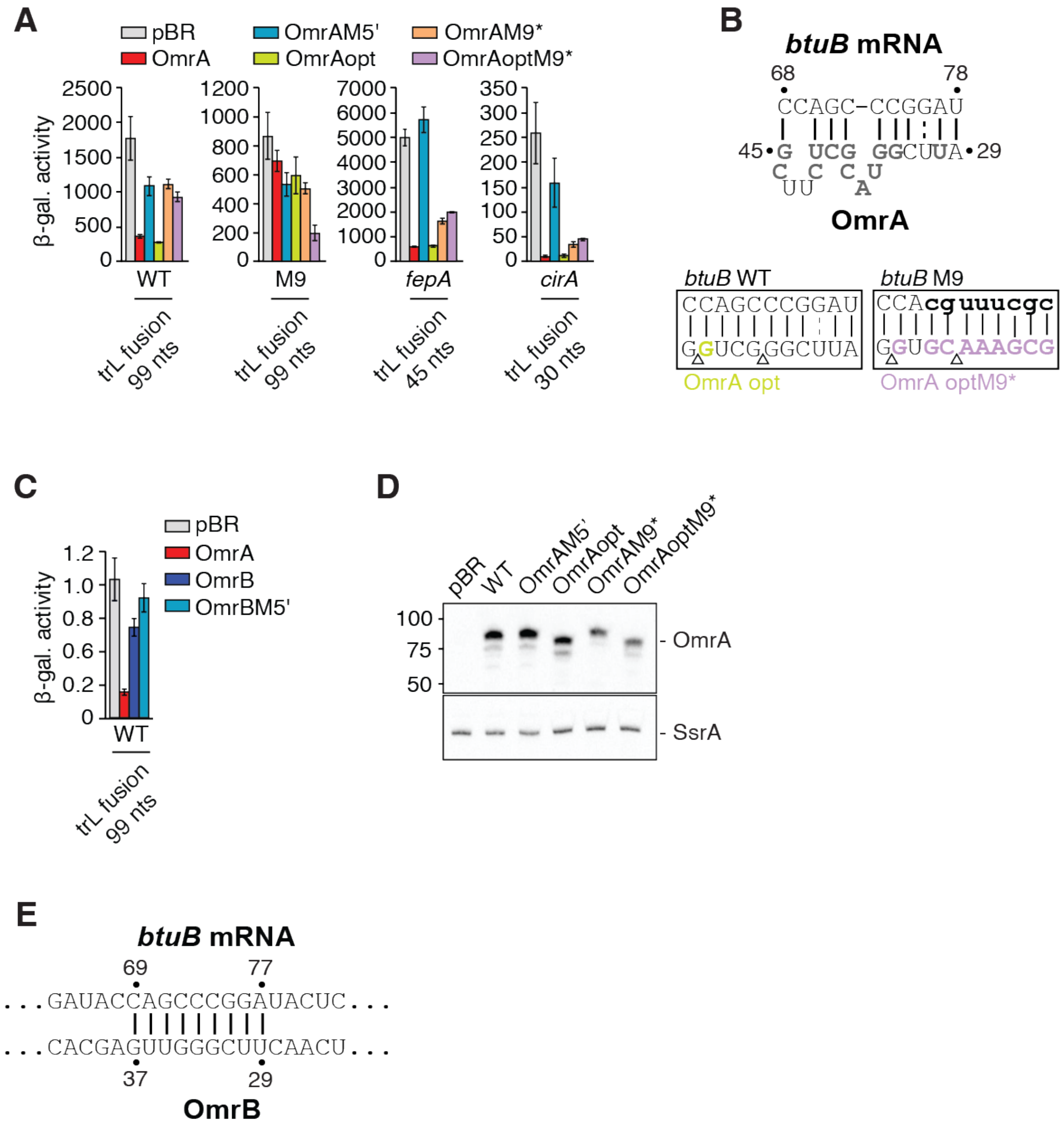

Supplementary Figure S6. Sequence changes in the 5' end and central region of OmrA modulate *btuB* regulation. (A) Beta-galactosidase assays of BtuB99-LacZ (WT and M9 mutant), FepA45-LacZ and CirA30-LacZ translational fusions upon overproduction of different OmrA variants. The β-galactosidase average values (in Miller units) and the standard deviations were obtained from three independent experiments. All fusions are expressed from a $P_{LtetO-1}$ constitutively expressed promoter. **(B)** Predicted OmrA-*btuB* interactions are shown for key sRNA-mRNA base-pairs for the WT and OmrAopt, as well

as for *btuB* M9 mutant and OmrAoptM9*. Lowercase and colored nucleotides represent mutations introduced in *btuB* M9 and OmrA, respectively; deletion of nts in OmrAopt is indicated by the triangles. **(C)** Beta-galactosidase assays of WT $BtuB_{99}$-LacZ translational fusion upon overproduction of OmrA (WT) and OmrB (WT and M5' variant) sRNAs. **(D)** Northern blot analysis of levels of OmrA variants using RNA extracted from cell cultures used for corresponding ß-galactosidase assays. Detection of the SsrA RNA was used as a loading control. **(E)** IntaRNA predicted interaction between OmrB and the first 100 nts of *btuB* coding region.

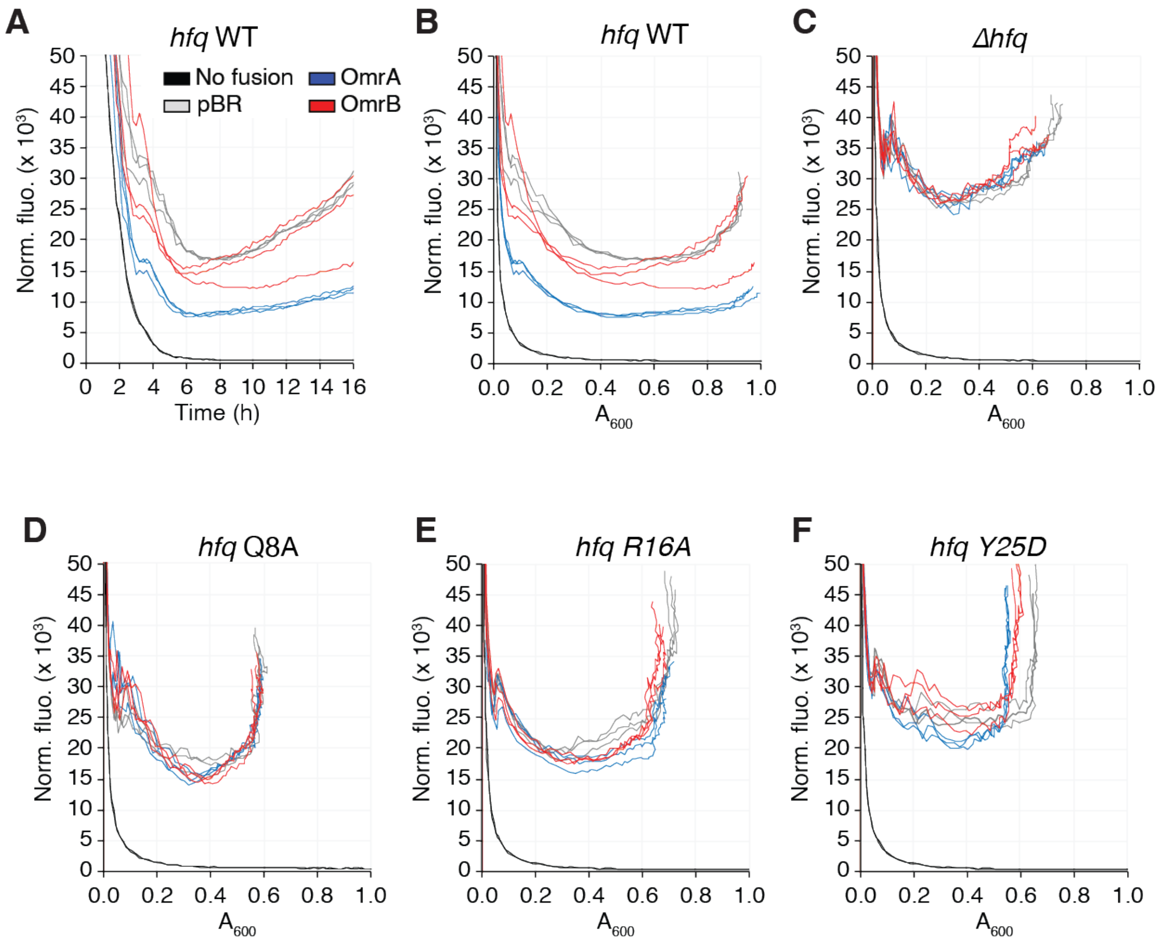

Supplementary Figure S7. Fluorescence assays using the $BtuB_{210}$-mScarlet fusion. The normalized fluorescence is shown versus time **(A)** or versus the absorbance at 600 nm **(B)** for the WT strain, and only as a function of absorbance for the other strains **(C-F)** Experiments were performed when overexpressing OmrA, OmrB or when using the empty vector (pBR). A control was performed as a strain without the fluorescent construct transformed with the blank vector (no fusion). Experiments were performed in the WT strain (*hfq* WT) **(A)**, Δ*hfq* **(B)**, *hfq* Q8A **(C)**, *hfq* R16A **(D)** and *hfq* Y25D **(E)**. These raw data were used to calculate the relative FPA (fluorescence per absorbance) shown in Figure 5B.

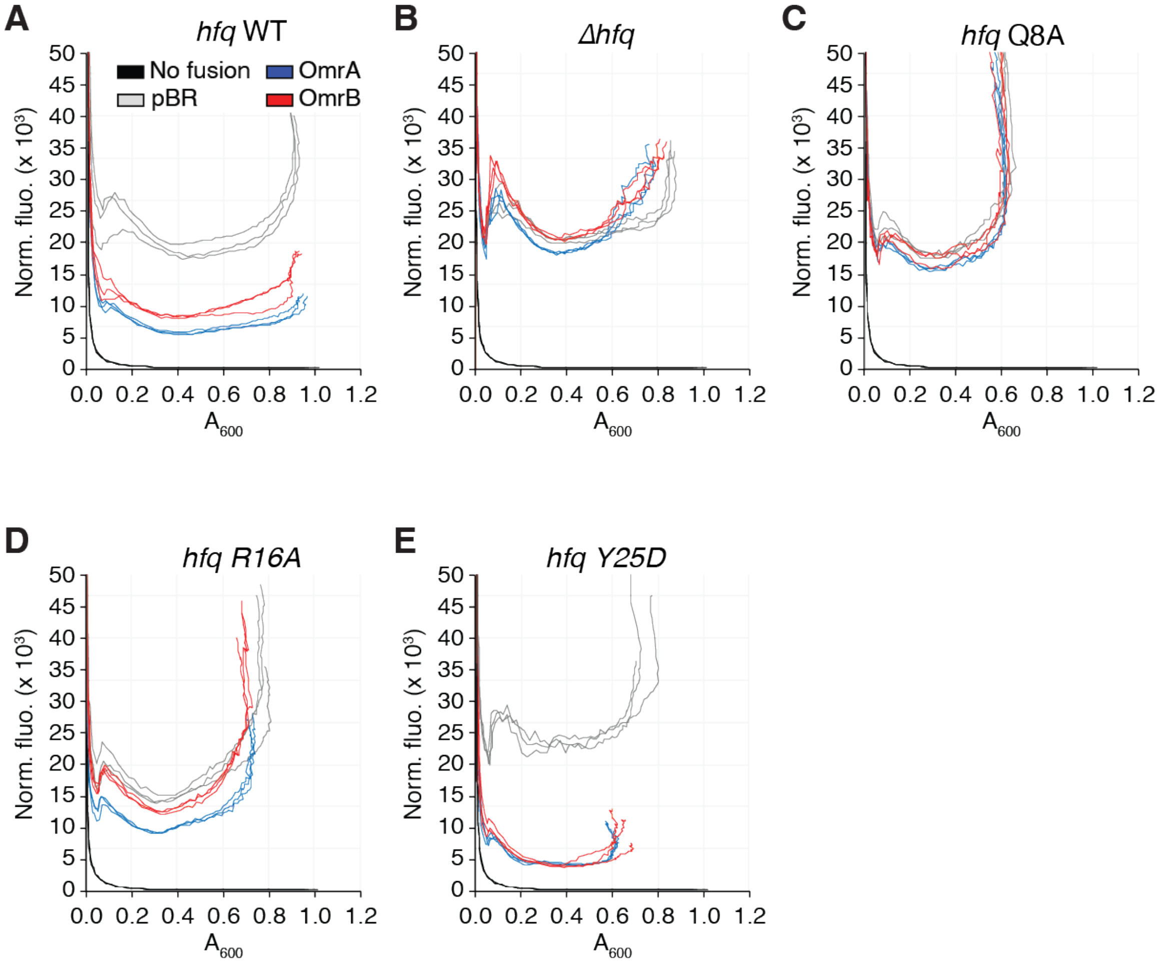

Supplementary Figure S8. Fluorescence assays using the OmpR_{717}-mScarlet fusion. **(A-E)** Experiments were performed when overexpressing OmrA, OmrB or when using the empty vector (pBR). The no fusion control was as in Supplementary Figure S7. Experiments were performed in the WT strain (*hfq* WT) **(A)**, Δ*hfq* **(B)**, *hfq* Q8A **(C)**, *hfq* R16A **(D)** and *hfq* Y25D **(E)**. These raw data were used to calculate the relative FPA (fluorescence per absorbance) shown in Figure 5C.

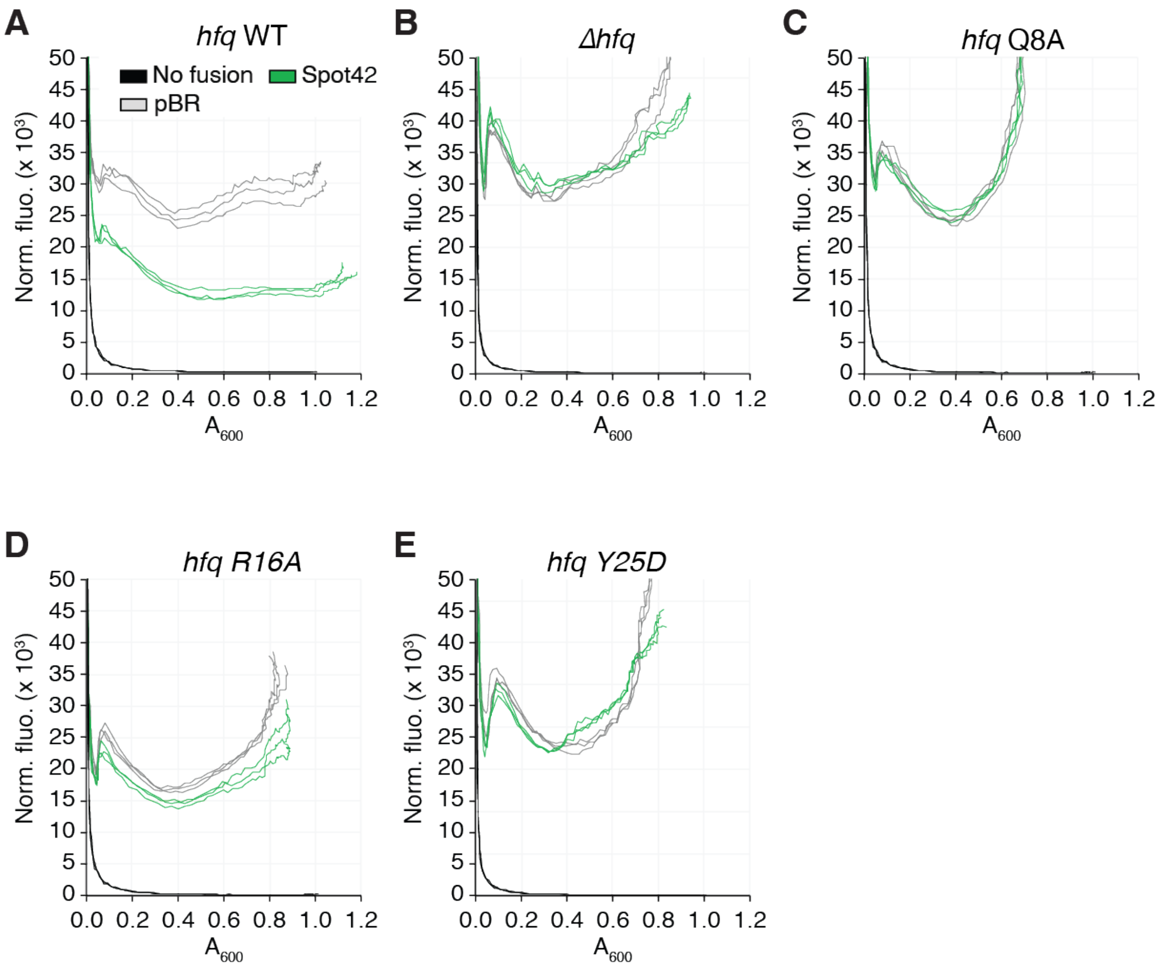

Supplementary Figure S9. Fluorescence assays using the SdhC$_{39}$-mScarlet fusion. (A-E) Experiments were performed when overexpressing Spot42 or when using the empty vector (pBR). The no fusion control was as in Supplementary Figure S7. Experiments were performed in the WT strain (*hfq* WT) **(A)**, Δ*hfq* **(B)**, *hfq* Q8A **(C)**, *hfq* R16A **(D)** and *hfq* Y25D **(E)**. These raw data were used to calculate the relative FPA (fluorescence per absorbance) shown in Figure 5D.

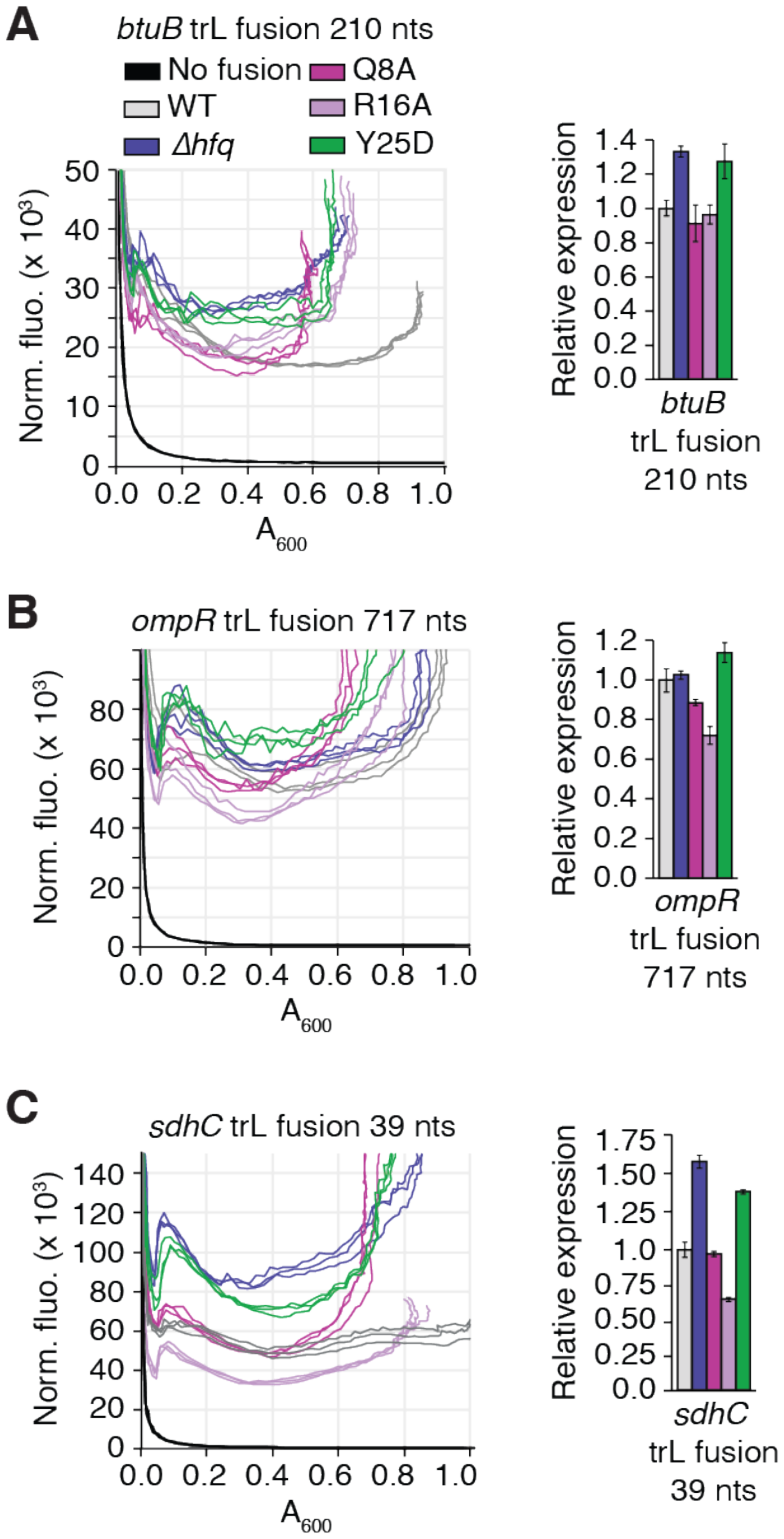

Supplementary Figure S10. The fluorescence of $BtuB_{210}$-mScarlet (A), $OmpR_{717}$-mScarlet (B) and $SdhC_{39}$-mScarlet (C) fusions was measured in different *hfq*

backgrounds, in strains transformed with the pBRplac empty vector. Data are from the datasets shown in Supplementary Figure S7 (*btuB*, **A**), S5 (*ompR*, **B**), and S6 (*sdhC*, **C**). The corresponding relative expression diagrams (right panels) were prepared as in Supplementary Figure S5.

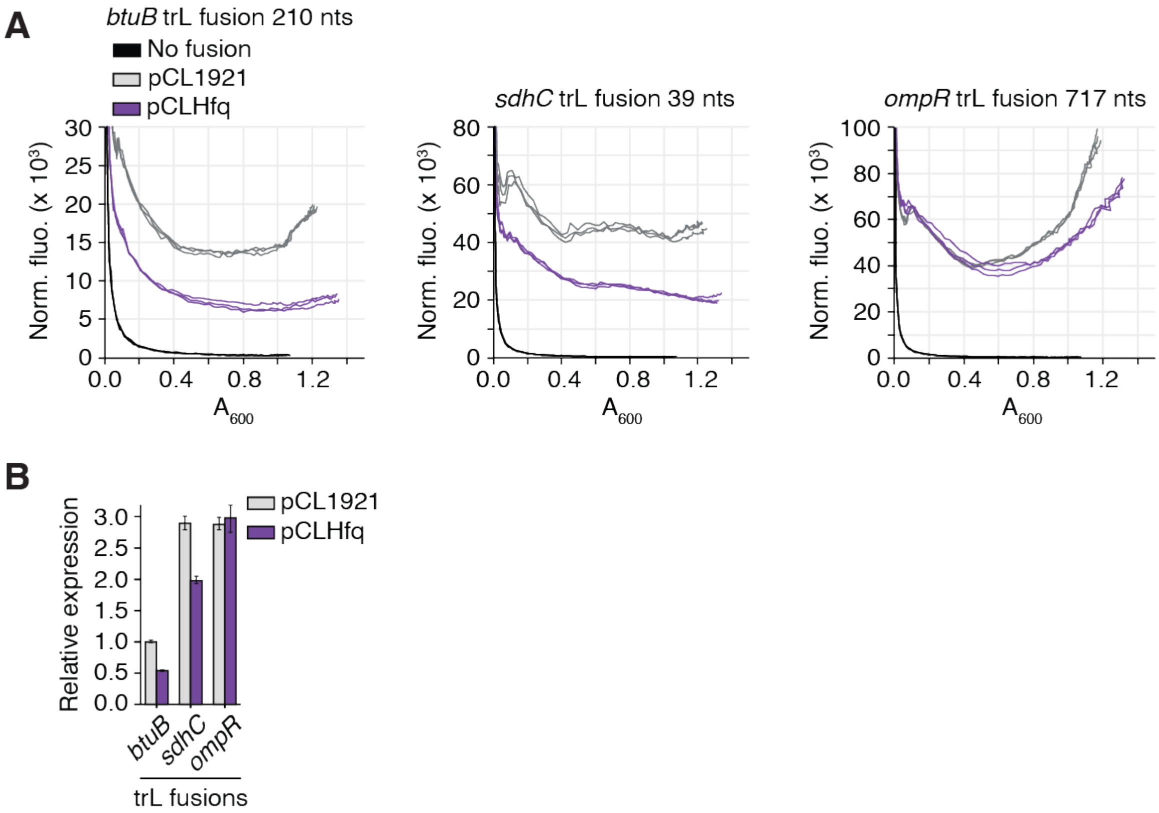

Supplementary Figure S11. Hfq overproduction represses *btuB* and *sdhC* expression. **(A)** Graphic representation is as in Supplementary Figure S5. **(B)** The fluorescence of the $BtuB_{210}$-, $OmpR_{717}$- and $SdhC_{39}$-mScarlet was measured in cells transformed with a plasmid overproducing Hfq (pCLHfq) or the corresponding empty vector (pCL1921) in triplicates.

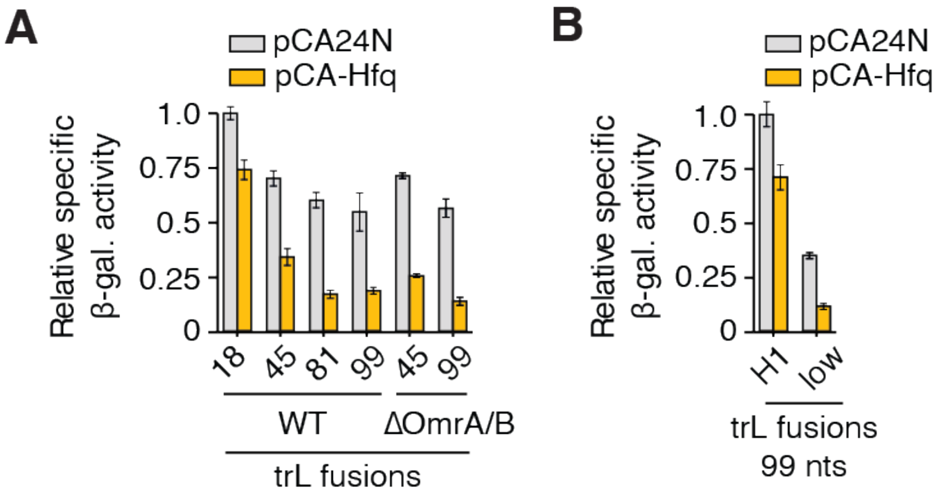

Supplementary Figure S12. Repression of the BtuB-LacZ fusions upon the Hfq overexpression as a function of *btuB* ORF length. (A) β-galactosidase assays of BtuB-LacZ translational fusions without or with overexpression of Hfq. The number of nucleotides of *btuB* ORF is indicated for each construct. The expression of the $BtuB_{45}$- and $BtuB_{99}$-LacZ fusions was measured in both *omrAB*$^{+}$ and *omrAB*$^{-}$ strains. **(B)** β-galactosidase assays of BtuB-LacZ translational fusions without or with overexpression of Hfq. Constructs either contain the H1 mutant or were expressed from a low expression promoter (low). For both panels, strains were grown in LB-Cam supplemented with 50 µM IPTG.

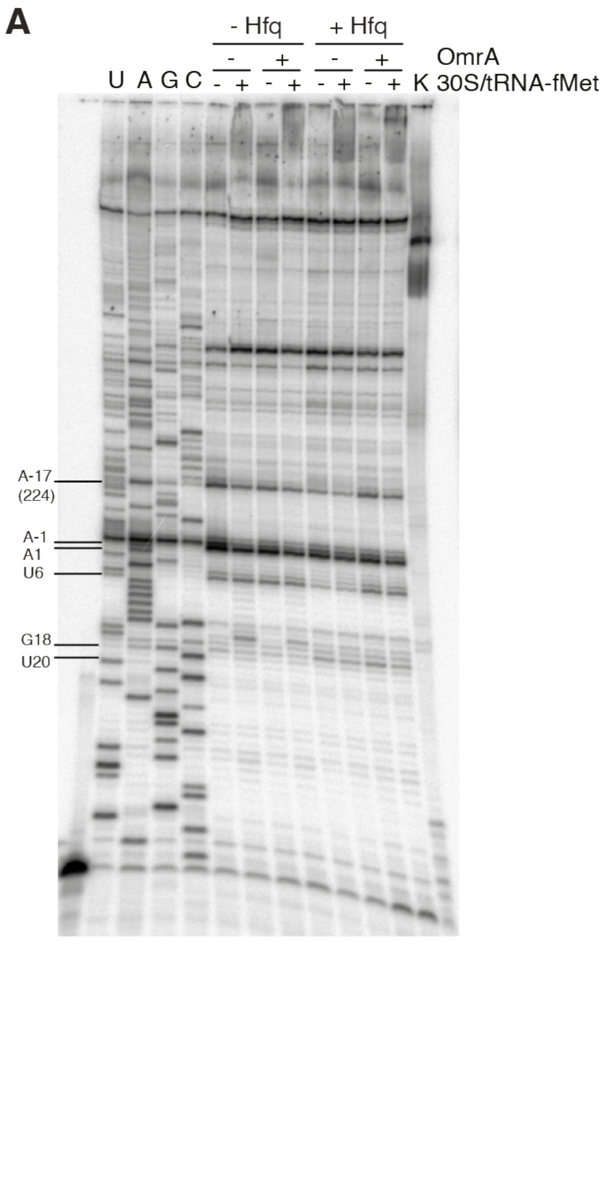

Supplementary Figure S13. Toeprint assays using the WT and the H1 mutant show that 30S binding is modulated. Toeprint assays monitoring the effects of Hfq and OmrA on the formation of the translation initiation complex with the WT **(A)** and the H1 mutant **(B)** variants of the *btuB* RNA. Experiments were performed in the absence or presence of Hfq, OmrA or 30S/tRNA-fMet. Lanes U, A, G and C represent sequencing ladders. K represents a result from an unrelated control experiment.

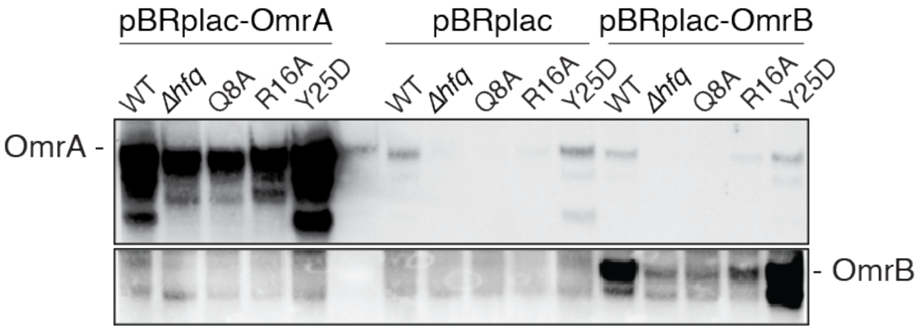

Supplementary Figure S14. Northern blot analysis of levels of OmrA and OmrB. Shown are the same Northern blots as in Figure 5E, with a longer acquisition time, so that the chromosomally-expressed OmrA sRNA can now be detected.

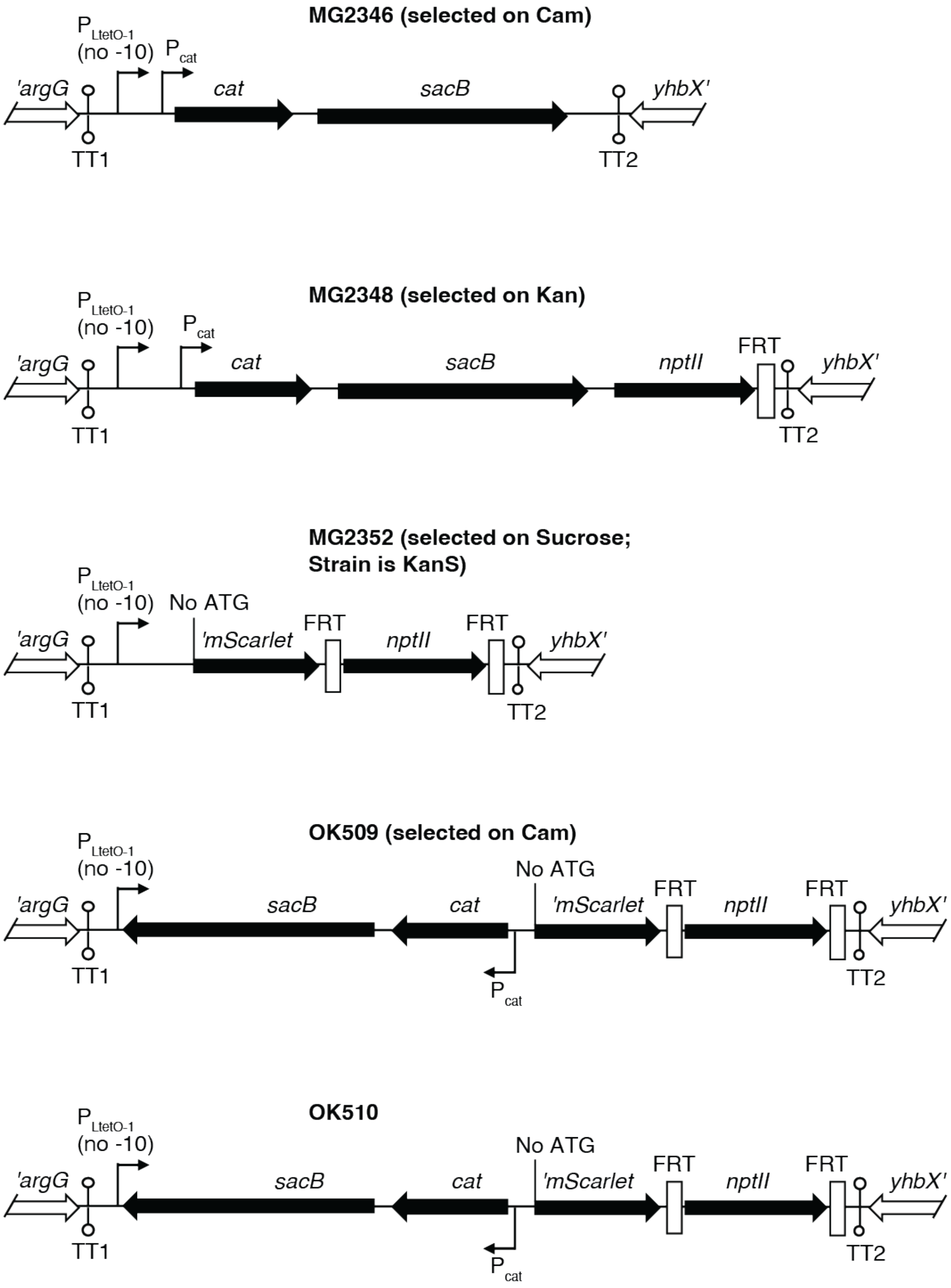

Supplementary Figure S15. Schematics of the different steps leading to the OK510 strain used for the construction of mScarlet fusions by recombineering. Shown is the genetic organization of the *argG-yhbX* intergenic region in the different intermediate strains. See supplementary text for details.

Table S1. Strains and plasmids used in this study.

Strain name	Characteristics	References
MG1655	E. coli reference strain for this study	From F. Blattner's lab
DJ480	MG1655 *ΔlacX74*	D. Jin, NIH
DJ624	MG1655 *ΔlacX74, mal*::*lacI*^{q}	D. Jin, NIH, used as 'no fusion' control strain in Fig. 5
EM1055	MG1655 *ΔlacX74*	(19) Same genotype as DJ480, used in Fig. 1
EM1264	MG1055 *hfq*::*cat*	(20) Used in Fig. 1
EM1377	EM1055 *rne131 zce-726*::*Tn10*	(20) Used in Fig. 1
PM1205	MG1655 *mal*::*lacI*^{q} *ΔaraBAD araC+ mini-λ-Tet lacI'* ::P_{BAD}-*cat-sacB-lacZ*	(8)
BTU1	PM1205 *lacI'*::P_{BAD}-BtuB$_{-240+18}$-LacZ$_{+28}$	(21) Used in Fig. 2 and S3
BTU8	PM1205 *lacI'*::P_{BAD}-BtuB$_{-240+63}$-LacZ$_{+28}$	This study, recombineering in PM1205, used in Fig. 2 and S3
BTU7	PM1205 *lacI'*::P_{BAD}-BtuB$_{-240+81}$-LacZ$_{+28}$	This study, recombineering in PM1205, used in Fig. 2 and S3
BTU20	PM1205 *lacI'*::P_{BAD}-BtuB$_{-240+81}$EP1-LacZ$_{+28}$	This study, recombineering in PM1205, used in Fig. 2
BTU21	PM1205 *lacI'*::P_{BAD}-BtuB$_{-240+81}$EP2-LacZ$_{+28}$	This study, recombineering in PM1205, used in Fig. 2
BTU22	PM1205 *lacI'*::P_{BAD}-BtuB$_{-240+81}$EP3-LacZ$_{+28}$	This study, recombineering in PM1205, used in Fig. 2
BTU6	PM1205 *lacI'*::P_{BAD}-BtuB$_{-240+120}$-LacZ$_{+28}$	This study, recombineering in PM1205, used in Fig. 2 and S3
BTU5	PM1205 *lacI'*::P_{BAD}-BtuB$_{-240+210}$-LacZ$_{+28}$	This study, recombineering in PM1205, used in Fig. 2 and S3
BTU4	PM1205 *lacI'*::P_{BAD}-BtuB$_{-240+420}$-LacZ$_{+28}$	This study, recombineering in PM1205, used in Fig. 2 and S3
BTU2	PM1205 *lacI'*::P_{BAD}-*btuB*$_{-240+18}$-*lacZ*$_{-38}$	(19) Used in Fig. 2 and S3
BTU13	PM1205 *lacI'* ::P_{BAD}-*btuB*$_{-240+81}$-*lacZ*$_{-38}$	This study, recombineering in PM1205, used in Fig. 2, 3A and 3B, and S3
BTU14	PM1205 *lacI'*::P_{BAD}-*btuB*$_{-240+45}$-*lacZ*$_{-38}$	This study, recombineering in PM1205, used in Fig. 2 and S3
BTU11	PM1205 *lacI'*::P_{BAD}-*btuB*$_{-240+210}$-*lacZ*$_{-38}$	This study, recombineering in PM1205, used in Fig. 2, 3A and 3B, and S3
BTU10	PM1205 *lacI'*::P_{BAD}-*btuB*$_{-240+420}$-*lacZ*$_{-38}$	This study, recombineering in PM1205, used in Fig. 2 and S3
DJS2604	MG1655 *Δhfq*::*cat-sacB, purA*::FRT-*nptII*-FRT	D. Schu, unpublished
DJS2609	MG1655 *hfq WT*	D. Schu, unpublished
DJS2927	MG1655 *Δhfq*	D. Schu, unpublished
HfrG6Δ*lac*12RevES191 *rne131 zce-726*::Tn*10*	An *rne131* donor strain	M. Dreyfus, provided by Eliane Hajnsdorf
JJ0015	MG1508 *mhpR*-$P_{LtetO-1}$-FepA$_{-173+45}$-LacZ$_{+28}$ *Δmini-λ-Tet ΔomrAB::nptI*	(22) Used in Fig. 4E and S6A
JJ360	MG1508 *mhpR*-$P_{LtetO-1}$-BtuB$_{-240+45}$-LacZ$_{+28}$*Δmini-λ-Tet*	This study; recombineering in MG1508
JJ389	MG1508 *mhpR*-$P_{LtetO-1}$-FepA$_{-146+45}$-LacZ$_{+28}$*Δmini-λ-Tet*	Lab collection (strain from J. Jagodnik), used in Fig. S5C
JJ415	MG1508 *mhpR*-$P_{LtetO-1}$-BtuB$_{-240+81}$-LacZ$_{+28}$*Δmini-λ-Tet*	This study; recombineering in MG1508
JJ416	MG1508 *mhpR*-$P_{LtetO-1}$-BtuB$_{-240+99}$-LacZ$_{+28}$*Δmini-λ-Tet*	This study; recombineering in MG1508, used in Fig. 4F-G, 6B, and S4A and S4D
JJ425	MG1508 *mhpR*-$P_{LtetO-1}$-BtuBM9$_{-240+99}$-LacZ$_{+28}$ *Δmini-λ-Tet*	This study; recombineering in MG1508, used in Fig. 4G and S4D

JJ426	JJ416 *ΔomrAB*::*nptI*	This study; JJ416 + P1 (MG1003), used in Fig. 4B-G, S6A, S6C and S6D
JJ427	JJ425 *ΔomrAB*::*nptI*	This study; JJ425 + P1 (MG1003), used in Fig. 4B, 4G and S6A
JW0334	BW25113 *ΔlacY*::FRT-*nptII*-FRT	Keio collection (11)
JW1087	BW25113 *ΔptsG*::FRT-*nptII*-FRT	Keio collection (11)
JW3140	BW25113 *ΔargG*::FRT-*nptII*-FRT	Keio collection (11)
JW5851	BW25113 *Δpnp*::FRT-*nptII*-FRT	Keio collection (11)
KK2560	MG1655 *hfqQ8A*	D. Schu, unpublished
KK2561	MG1655 *hfqR16A*	D. Schu, unpublished
KK2562	MG1655 *hfqY25D*	D. Schu, unpublished
MG1001	DJ480 *ΔomrA*::*nptI Δmini-λ-Tet*	This study; recombineering in NM300
MG1002	DJ480 *ΔomrB*::*nptI Δmini-λ-Tet*	This study; recombineering in NM300
MG1003	DJ480 *ΔomrAB*::*nptI Δmini-λ-Tet*	(10)
MG1099	DJ624 *ΔomrAB*::*nptI*	(10)
MG1432	DJ624 *mini-λ-Tet*	M. Guillier, unpublished.
MG1508	MG1655 *mal*::*lacI^q*, *mini-λ-Tet, mhpR*-P$_{LtetO-1}$-*cat-sacB-lacZ*	(9)
MG1582	MG1508 *mhpR*-P$_{LtetO-1}$-*cirA$_{-160+30}$*-*lacZ$_{+28}$* *Δmini-λ-Tet*	This study; recombineering in MG1508
MG2288	MG1582 *ΔomrAB*::*nptI*	This study; MG1582 + P1 (MG1003), used in Fig. 4E and S6A
MG2346	MG1432 *argG*-[TT1-P$_{LtetO-1}$(no -10)-*cat-sacB*-TT2]-*yhbX*	This study
MG2348	MG2346 *argG*-[TT1-P$_{LtetO-1}$(no -10)-*cat-sacB-nptII*-FRT-TT2]-*yhbX*	This study
MG2352	MG2348 *argG*-[TT1-P$_{LtetO-1}$(no -10)-*'mScarlet*(no ATG)-FRT-*nptII*-FRT-TT2]-*yhbX*	This study
MG2389	OK819 *ΔomrAB*::*nptI*	This study; OK819 + P1 (MG1003), used in Fig. 4C
NM300	DJ480 *mini-λ-Tet*	N. Majdalani, NIH
OK509	MG2352 *argG*-[TT1-P$_{LtetO-1}$(no -10)- *sacB-cat-'mScarlet*(no ATG)-FRT-*nptII*-FRT-TT2]-*yhbX*	This study; recombineering: *cat-sacB* cassette recombined into MG2352
OK510	MG1432 *argG*-[TT1-P$_{LtetO-1}$(no -10)-*sacB-cat-'mScarlet*(no ATG)-FRT-*nptII*-FRT-TT2]-*yhbX*	This study; MG1432 + P1 (OK509)
OK523	DJ624 *ΔargG*::FRT-*nptII*-FRT	This study; DJ624 + P1 (JW3140)
OK528	OK510 *argG*-[TT1-P$_{LtetO-1}$-SdhC$_{-222+39}$-mScarlet$_{+4}$-FRT-*nptII*-FRT-TT2]-*yhbX, Δmini-λ-Tet*	This study; recombineering in OK510
OK529	OK510 *argG*-[TT1-P$_{LtetO-1}$-OmpR$_{-35+717}$-mScarlet$_{+4}$-FRT-*nptII*-FRT-TT2]-*yhbX, Δmini-λ-Tet*	This study; recombineering in OK510
OK530	OK523 *ΔargG ::*FRT	This study; elimination of *nptII* in OK523 *via* Flp-FRT recombination
OK560	OK528 *argG*-[TT1-P$_{LtetO-1}$-SdhC$_{-222+39}$-mScarlet$_{+4}$-FRT-TT2]-*yhbX*	This study; elimination of *nptII* in OK528 *via* Flp-FRT recombination
OK561	OK529 *argG*-[TT1-P$_{LtetO-1}$-OmpR$_{-35+717}$-mScarlet$_{+4}$- FRT-TT2]-*yhbX*	This study; elimination of *nptII* in OK529 *via* Flp-FRT recombination
OK564	OK530 *Δhfq*::*cat-sacB, purA*::FRT-*nptII*-FRT	This study; OK530 + P1 (DJS2604)
OK572	OK510 *argG*-[TT1-P$_{LtetO-1}$-BtuB$_{-240+210}$-mScarlet$_{+4}$-FRT-*nptII*-FRT-TT2]-*yhbX, Δmini-λ-Tet*	This study; recombineering in OK510
OK577	OK572 *argG*-[TT1-P$_{LtetO-1}$-BtuB$_{-240+210}$-mScarlet$_{+4}$-FRT-TT2]-*yhbX*	This study; elimination of *nptII* in OK572 *via* Flp-FRT recombination
OK581	OK564 *hfq WT*	This study; OK564 + P1 (DJS2609) (purine prototroph selection)
OK582	OK564 *Δhfq*	This study; OK564 + P1 (DJS2927) (purine prototroph selection)
OK583	OK564 *hfqQ8A*	This study; OK564 + P1 (KK2560) (purine prototroph selection)

OK584	OK564 *hfqR16A*	This study; OK564 + P1 (KK2561) (purine prototroph selection)
OK585	OK564 *hfqY25D*	This study; OK564 + P1 (KK2562) (Selecting for SucrR)
OK586	OK581 *argG*-[TT1-P$_{LtetO-1}$-BtuB$_{-240+210}$-mScarlet$_{+4}$-FRT-TT2]-*yhbX*	This study; OK581 + P1 (OK577) (arginine prototroph selection), used in Fig. 5A,B,E, and S5A,B,D,F, S7, S10 and S11
OK587	OK582 *argG*-[TT1-P$_{LtetO-1}$-BtuB$_{-240+210}$-mScarlet$_{+4}$-FRT-TT2]-*yhbX*	This study; OK582 + P1 (OK577) (arginine prototroph selection), used in Fig. 5B and 5E, and S7 and S10
OK588	OK583 *argG*-[TT1-P$_{LtetO-1}$-BtuB$_{-240+210}$-mScarlet$_{+4}$-FRT-TT2]-*yhbX*	This study; OK583 + P1 (OK577) (arginine prototroph selection), used in Fig. 5B and 5E, and S7 and S10
OK589	OK584 *argG*-[TT1-P$_{LtetO-1}$-BtuB$_{-240+210}$-mScarlet$_{+4}$-FRT-TT2]-*yhbX*	This study; OK584 + P1 (OK577) (arginine prototroph selection), used in Fig. 5B and 5E, and S7 and S10
OK590	OK585 *argG*-[TT1-P$_{LtetO-1}$-BtuB$_{-240+210}$-mScarlet$_{+4}$-FRT-TT2]-*yhbX*	This study; OK585 + P1 (OK577) (arginine prototroph selection), used in Fig. 5B and 5E, and S7 and S10
OK596	OK581 *argG*-[TT1-P$_{LtetO-1}$-SdhC$_{-222+39}$-mScarlet$_{+4}$-FRT- TT2]-*yhbX*	This study; OK581 + P1 (OK560) (arginine prototroph selection), used in Fig. 5D and 5F, and S9, S10 and S11
OK597	OK582 *argG*-[TT1-P$_{LtetO-1}$-SdhC$_{-222+39}$-mScarlet$_{+4}$-FRT- TT2]-*yhbX*	This study; OK582 + P1 (OK560) (arginine prototroph selection), used in Fig. 5D and 5F, and S9 and S10
OK598	OK583 *argG*-[TT1-P$_{LtetO-1}$-SdhC$_{-222+39}$-mScarlet$_{+4}$-FRT- TT2]-*yhbX*	This study; OK583 + P1 (OK560) (arginine prototroph selection), used in Fig. 5D and 5F, and S9 and S10
OK599	OK584 *argG*-[TT1-P$_{LtetO-1}$-SdhC$_{-222+39}$-mScarlet$_{+4}$-FRT- TT2]-*yhbX*	This study; OK584 + P1 (OK560) (arginine prototroph selection), used in Fig. 5D and 5F, and S9 and S10
OK600	OK585 *argG*-[TT1-P$_{LtetO-1}$-SdhC$_{-222+39}$-mScarlet$_{+4}$-FRT- TT2]-*yhbX*	This study; OK585 + P1 (OK560) (arginine prototroph selection), used in Fig. 5D and 5F, and S9 and S10
OK601	OK581 *argG*-[TT1-P$_{LtetO-1}$-OmpR$_{-35+717}$-mScarlet$_{+4}$- FRT-TT2]-*yhbX*	This study; OK581 + P1 (OK561) (arginine prototroph selection), used in Fig. 5C, and S8, S10 and S11
OK602	OK582 *argG*-[TT1-P$_{LtetO-1}$-OmpR$_{-35+717}$-mScarlet$_{+4}$- FRT-TT2]-*yhbX*	This study; OK582 + P1 (OK561) (arginine prototroph selection), used in Fig. 5C, and S8 and S10
OK603	OK583 *argG*-[TT1-P$_{LtetO-1}$-OmpR$_{-35+717}$-mScarlet$_{+4}$- FRT-TT2]-*yhbX*	This study; OK583 + P1 (OK561) (arginine prototroph selection), used in Fig. 5C, and S8 and S10
OK604	OK584 *argG*-[TT1-P$_{LtetO-1}$-OmpR$_{-35+717}$-mScarlet$_{+4}$- FRT-TT2]-*yhbX*	This study; OK584 + P1 (OK561) (arginine prototroph selection), used in Fig. 5C, and S8 and S10
OK605	OK585 *argG*-[TT1-P$_{LtetO-1}$-OmpR$_{-35+717}$-mScarlet$_{+4}$- FRT-TT2]-*yhbX*	This study; OK585 + P1 (OK561) (arginine prototroph selection), used in Fig. 5C, and S8 and S10
OK615	JJ416 *ΔomrA*::*nptI*	This study; JJ416 + P1 (MG1001), used in Fig. 4F-G
OK616	JJ416 *ΔomrB*::*nptI*	This study; JJ416 + P1 (MG1002), used in Fig. 4F-G
OK617	JJ425 *ΔomrA*::*nptI*	This study; JJ425 + P1 (MG1001), used in Fig. 4G
OK618	JJ425 *ΔomrB*::*nptI*	This study; JJ425 + P1 (MG1002), used in Fig. 4G
OK627	OK586 *Δpnp*::FRT-*nptII*-FRT	This study, OK586 + P1 (JW5851), used in Fig. S5A,B,E,F
OK632	MG1508 *mhpR*-P$_{LtetO-1}$-BtuB$_{-240+99}$mutH1-lacZ$_{+28}$*Δmini-λ-Tet*	This study; recombineering in MG1508, used in Fig. 6B

OK737	MG1508 *mhpR*-lowP$_{LtetO-1}$-BtuB$_{-240+99}$-lacZ$_{+28}$*Δmini-λ-Tet*	This study; recombineering in MG1508, used in Fig. 6B
OK750	MG1508 *mhpR*-P$_{LtetO-1}$-*btuB*$_{-240+210}$-*lacZ*$_{-38}$ *Δmini-λ-Tet*	This study; recombineering in MG1508, used in Fig. 3C
OK751	MG1508 *mhpR*-P$_{LtetO-1}$-*btuB*$_{-240+420}$-*lacZ*$_{-38}$ *Δmini-λ-Tet*	This study; recombineering in MG1508, used in Fig. 3C
OK756	DJ624 *rhoR66S*	(23)
OK762	OK756 *mhpR*-P$_{LtetO-1}$-*btuB*$_{-240+210}$-*lacZ*$_{-38}$	This study; OK756 + P1(OK750), used in Fig. 3C
OK763	OK756 *mhpR*-P$_{LtetO-1}$-*btuB*$_{-240+420}$-*lacZ*$_{-38}$	This study; OK756 + P1(OK751), used in Fig. 3C
OK764	MG1508 *mhpR*-P$_{LtetO-1}$-*btuB*$_{-240+81}$-*lacZ*$_{-38}$ *Δmini-λ-Tet*	This study; recombineering in MG1508, used in Fig. 3C
OK819	MG1508 *mhpR*-P$_{LtetO-1}$-*btuBM11*$_{-240+99}$-*lacZ*$_{+28}$ *Δmini-λ-Tet*	This study; recombineering in MG1508
OK833	MG1508 *mhpR*-P$_{LtetO-1}$-BtuB$_{-240+99}$EP1-lacZ$_{+28}$ *Δmini-λ-Tet*	This study; recombineering in MG1508, used in Fig. S4A
OK835	MG1508 *mhpR*-P$_{LtetO-1}$-BtuB$_{-240+99}$EP3-lacZ$_{+28}$ *Δmini-λ-Tet*	This study; recombineering in MG1508, used in Fig. S4A
OK836	MG1508 *mhpR*-P$_{LtetO-1}$-BtuB$_{-240+18}$-lacZ$_{+28}$ *Δmini-λ-Tet*	This study; recombineering in MG1508
OK837	DJ624 *zce-726*::Tn*10* *rne131*	This study; DJ624 + P1 (HfrG6Δ*lac*12RevES191 *rne131* *zce-726*::Tn*10*)
OK838	DJ624 *ΔptsG*::FRT-*nptII*-FRT *rne131*	This study; OK837 + P1(JW1087)
OK843	OK756 *mhpR*-P$_{LtetO-1}$-*btuB*$_{-240+81}$-*lacZ*$_{-38}$	This study; OK756 + P1(OK764), used in Fig. 3C
OK852	MG1508 *mhpR*-P$_{LtetO-1}$-*btuB*$_{-240+210}$EP1-*lacZ*$_{-38}$ *Δmini-λ-Tet*	This study; recombineering in MG1508, used in Fig. S4B
OK854	MG1508 *mhpR*-P$_{LtetO-1}$-*btuB*$_{-240+210}$EP3-*lacZ*$_{-38}$ *Δmini-λ-Tet*	This study; recombineering in MG1508, used in Fig. S4B
OK855	OK764 *ΔptsG*::FRT-*nptII*-FRT *rne131*	This study; OK764 + P1(OK838), *rne131* allele checked by PCR, used in Fig. S4C
OK856	OK764 *ΔptsG*::FRT-*nptII*-FRT	This study; OK764 + P1(OK838), *rne*$^{+}$ allele checked by PCR, used in Fig. S4C
OK857	OK750 *ΔptsG*::FRT-*nptII*-FRT *rne131*	This study; OK750 + P1(OK838), *rne131* allele checked by PCR, used in Fig. S4C
OK858	OK750 *ΔptsG*::FRT-*nptII*-FRT	This study; OK750 + P1(OK838), *rne*$^{+}$ allele checked by PCR, used in Fig. S4C
OK859	OK751 *ΔptsG*::FRT-*nptII*-FRT *rne131*	This study; OK751 + P1(OK838), *rne131* allele checked by PCR, used in Fig. S4C
OK860	OK751 *ΔptsG*::FRT-*nptII*-FRT	This study; OK751 + P1(OK838), *rne*$^{+}$ allele checked by PCR, used in Fig. S4C
OK865	MG1508 *ΔlacY::*FRT-*nptII*-FRT *Δmini-λ-Tet*	This study; recombineering in MG1508 (selecting for KanR)
OK866	OK865 *ΔlacY::*FRT	This study; elimination of *nptII* in OK865 *via* Flp-FRT recombination
OK868	DJ624 *mini-λ-Tet, mhpR*-P$_{LtetO-1}$-*cat-sacB-lacZ* *ΔlacY*::FRT	This study; MG1432 + P1 (OK866)
OK870	OK868 *mhpR*-P$_{LtetO-1}$-BtuB$_{-240+18}$-lacZ$_{+28}$ *Δmini-λ-Tet*	This study; recombineering in OK868, used in Fig. S12A.
OK871	OK868 *mhpR*-P$_{LtetO-1}$-BtuB$_{-240+45}$-lacZ$_{+28}$ *Δmini-λ-Tet*	This study; recombineering in OK868, used in Fig. S12A.
OK872	OK868 *mhpR*-P$_{LtetO-1}$-BtuB$_{-240+81}$-lacZ$_{+28}$ *Δmini-λ-Tet*	This study; recombineering in OK868, used in Fig. S12A.

OK873	OK868 *mhpR*-$P_{LtetO-1}$-BtuB$_{-240+99}$-lacZ$_{+28}$ *Δmini-λ-Tet*	This study; recombineering in OK868, used in Fig. S12A.
OK874	OK868 *mhpR*-$P_{LtetO-1}$-BtuB$_{-240+99}$mutH1-lacZ$_{+28}$ *Δmini-λ-Tet*	This study; recombineering in OK868 , used in Fig. S12B.
OK875	OK868 *mhpR*-low$P_{LtetO-1}$-BtuB$_{-240+99}$-lacZ$_{+28}$ *Δmini-λ-Tet*	This study; recombineering in OK868, used in Fig. S12B.
OK886	OK871 *ΔomrAB*::*nptI*	This study; OK871 + P1 (MG1099) , used in Fig. S12A.
OK887	OK873 *ΔomrAB*::*nptI*	This study; OK873 + P1 (MG1099) , used in Fig. S12A.
**Plasmid**	**Characteristics**	**Use**
pNM12	AmpR, pBAD24 derivative	(24) pBAD vector control
pBAD-OmrA	*omrA* under P_{BAD} control in pNM12	(10) OmrA overproduction
pBAD-OmrB	*omrB* under P_{BAD} control in pNM12	(10) OmrB overproduction
pCP20	FLP^+, λ $cI857^+$, λ p_R Rep^{TS}, Amp^R, Cam^R	(12) *nptII* elimination *via* Flp-FRT recombination
pBRplac	Amp^R, Tet^R, $P_{LlacO-1}$ cloned into pBR322	(10) vector control
pOmrA	*omrA* under $P_{LlacO-1}$ control in pBRplac	(10) OmrA overproduction
pOmrAM5'	*omrAM5'* under $P_{LlacO-1}$ control in pBRplac<br>M5'= change of nt 3-6 of OmrA from CAGA to GAAC (*a.k.a. omrAmut2* in (10))	(10) OmrAM5' overproduction
pOmrAM9*	*omrAM9** under $P_{LlacO-1}$ control in pBRplac<br>M9 = change of nt 26-36 of OmrA from AUUCGGUACGC to GCGAAAUACCG	This study; OmrAM9* overproduction
pOmrAM11*	*omrAM11** under $P_{LlacO-1}$ control in pBRplac<br>M9 = change of nt 20-22 of OmrA from GAG to CUC	This study; OmrAM11* overproduction
pOmrAopt	*omrAopt* under $P_{LlacO-1}$ control in pBRplac<br>opt= change of nt 32-41 of OmrA from UACGCUCUUC to GCUG (*a.k.a. omrAmut8* in the lab stock)	This study; OmrAopt overproduction
pOmrAoptM9*	*omrAoptM9** under $P_{LlacO-1}$ control in pBRplac<br>optM9* = change of nt 26-41 of OmrA from AUUCGGUACGCUCUUC to GCGAAACGUG (*a.k.a. omrAmut8+9* in the lab stock, it is the compensatory change to *btuBM9* variant)	This study; OmrAoptM9* overproduction
pOmrB	*omrB* under $P_{LlacO-1}$ control in pBRplac	(10) OmrB overproduction
pSpot42	*spf* under $P_{LlacO-1}$ control in pBRplac	(25) Spot 42 overproduction
pCL1921	SpcR/StrR, pSC101 derivative	(26)
pCL*hfq*$^+$	*E. coli hfq* under its natural promoter control in pCL1921	(27)
pCA24N	CamR, $P_{T5\text{-}lac,}$ $lacI^q$,	(14), vector control used in Fig. S12
pCAS18	*E. coli rpsR* gene under control of $P_{T5\text{-}lac,}$ in pCA24N	M. Springer, unpublished
pCAS18n	*Nde*I site introduced at the *rpsR* start codon in pCAS18	This study; made by PCR mutagenesis
pHfq	*E. coli hfq* under P_{T7} control in pET11a-PL	(15)
pNK139	*E. coli hfq* under $P_{T5\text{-}lac}$ control in pCAS18n	This study; used in Fig. S12 (pCA-Hfq)

FRT is the Flp recombinase target site.
mini-λ-Tet is a λ prophage lacking replication and lytic functions. It provides Red functions required for recombination, and resistance to tetracycline (3).
mScarlet is a fluorescent reporter (28); the mScarlet-I gene was optimized for codon usage in *E. coli* (6).
nptI and *nptII* are kanamycin resistance ORFs originating from Tn903 and Tn5, respectively.
$P_{LtetO-1}$ and $P_{LlacO-1}$ are hybrid tetracycline and IPTG-inducible promoters, respectively (5).

Table S2. Oligonucleotides used in this study.

Name	Sequence	Use
**Strains construction: P_{BAD}-driven *lacZ* fusions**		
LB18	ACCTGACGCTTTTTATCGCAACTCTCTACTGTTTCTCCATGCCGGTC CTGTGAGTTAATAG	Forward primer for construction of P_{BAD}-*btuB-lacZ* transcriptional and translational fusions
LB66	TAACGCCAGGGTTTTCCCAGTCACGACGTTGTAAAACGACCTGTGCC CAAGCGGAAAATG	Reverse primer for BTU8 construction
LB67	TAACGCCAGGGTTTTCCCAGTCACGACGTTGTAAAACGACAGTATC CGGGCTGGTATCC	Reverse primer for BTU7 construction
LB75	TAACGCCAGGGTTTTCCCAGTCACGACGTTGTAAAACGACGCTGCGC GGCTGTTCAAAAC	Reverse primer for BTU6 construction
LB76	TAACGCCAGGGTTTTCCCAGTCACGACGTTGTAAAACGACCGGAAG ACGGCGCAGCA	Reverse primer for BTU5 construction
LB77	TAACGCCAGGGTTTTCCCAGTCACGACGTTGTAAAACGACGGAACC ATAAACAGCGGAGC	Reverse primer for BTU4 construction
LB78	GTGTGATAAAGAAAGTTAAAATGCCGGATCTGCCGTGACGGAACAC GC	Reverse primer for BTU14 construction
LB79	GTGTGATAAAGAAAGTTAAAATGCCGGATCAGTATCCGGGCTGGTA TCCTG	Reverse primer for BTU13 construction
LB80	GTGTGATAAAGAAAGTTAAAATGCCGGATCCGGAAGACGGCGCAGC A	Reverse primer for BTU11 construction
LB81	GTGTGATAAAGAAAGTTAAAATGCCGGATCGGAACCATAAACAGCG GAGC	Reverse primer for BTU10 construction
Stop 5'-3'	TAACGCCAGGGTTTTCCCAGTCACGACGTTGTAAAACGACCATAGC TGTTTCCTGTGTGATAAAGAAAGTTAAAATGCCGGATC	Reverse primer to include the stop codon sequence followed by *lacZ* translation initiation region and homology region within *lacZ* (construction of transcriptional fusions)
LB69	GGTTCTCATCATGCCGTAATATTGATG	Forward primer for EP1 mutant fusion construction
LB70	CATCAATATTACGGCATGATGAGAACC	Reverse primer for EP1 mutant fusion construction
LB71	GATGAAACCTGCCCGCATCCTTCTTCTATTG	Forward primer for EP2 mutant fusion construction
LB72	CAATAGAAGAAGGATGCGGGCAGGTTTCATC	Reverse primer for EP2 mutant fusion construction
LB73	CTTCTATTGTGGATCGAATACAATGATTAAAAAAGCTTCGC	Forward primer for EP3 mutant fusion construction
LB74	GCGAAGCTTTTTTAATCATTGTATTCGATCCACAATAGAAG	Reverse primer for EP3 mutant fusion construction
**Strains construction: $P_{LtetO-1}$-driven *lacZ* fusions**		
5'Ptet-cirA	GATAGAGATTGACATCCCTATCAGTGATAGAGATACTGAGCACAAT CAAAAAAGGCTGACAAATCAG	$P_{LtetO-1}$-CirA$_{-160+30}$-LacZ$_{+28}$ construction
3'cirA-lacZ	TAACGCCAGGGTTTTCCCAGTCACGACGTTGTAAAACGACGACCCG TACGAAAGGGTTCAAC	
5'PtetBtuB-240	CCCTATCAGTGATAGAGATACTGAGCACGCCGGTCCTGTGAGTTAA TAGGGAATCC	$P_{LtetO-1}$-BtuB$_{-240+210}$-mScarlet$_{+4}$ (or $P_{LtetO-1}$-BtuB$_{-240+99}$-LacZ$_{+28}$) construction
3'btuB45lacZrev	CCCAGTCACGACGTTGTAAAACGACTGCCGTGACGGAACACGCCGTC AGCAGCG	$P_{LtetO-1}$-BtuB$_{-240+45}$-LacZ$_{+28}$ construction
3'lacZ-btuB+81	CCCAGTCACGACGTTGTAAAACGACAGTATCCGGGCTGGTATCCTG TGCCC	$P_{LtetO-1}$-BtuB$_{-240+81}$-LacZ$_{+28}$ construction
3'lacZ-btuB+99	CCCAGTCACGACGTTGTAAAACGACGTTAGCAGTAACGACGAGAGT ATCCG	$P_{LtetO-1}$-BtuB$_{-240+99}$-LacZ$_{+28}$ construction
btuBmut9rev	CCCAGTCACGACGTTGTAAAACGACGTTAGCAGTAACGACGAGAGT GCGAAACGTGGTATCCTGTGCCCAAGCGG	$P_{LtetO-1}$-BtuBM9$_{-240+99}$-LacZ$_{+28}$ construction
AK51	CATATGGGGATTGGTGGCGACGAC	Amplification of *lacY* locus, Forward
AK52	CGGTAAGCCTTCGCACATATCGG	Amplification of *lacY* locus, Reverse
AK462	GTGGATGCTTTACAATGACCACCACCGCTTCGCTGC	$P_{LtetO-1}$-BtuB$_{-240+99}$mutH1-LacZ$_{+28}$ construction
AK675	GCCCGGATACTGAGGTCGTTACTGC	$P_{LtetO-1}$-BtuBM11$_{-240+99}$-LacZ$_{+28}$ construction
AK701	GGTTCTCATCATGCCGTAATATTGATG	*btuB* EP1 construction, forward
AK703	CCTTCTTCTATTGTGGATCGAATACAATGATTAAAAAAGC	*btuB* EP3 construction, forward

AK704	CACGACGTTGTAAAACGACCGAAGCTTTTTAATCATTGTAAAGC	$P_{LtetO-1}$-BtuB$_{-240+18}$-LacZ$_{+28}$ fusion construction
AK283	TGACACCATCGAATGGCGCTCCCTATCAGTGATAGAGATGGACATC CCTATC	low$P_{LtetO-1}$-BtuB$_{-240+99}$-LacZ$_{+28}$ construction (amplification using JJ416 strain as template)
Ptet-55-12For	CTCCCTATCAGTGATAGAGATTGACATCCCTATCAGTGATAGAG	Used to increase the length of the homology region with $P_{LtetO-1}$ for recombineering if needed (used also for the construction of the mScarlet fusions)
lacZ28-66rev	AACGCCAGGGTTTTCCCAGTCACGACGTTGTAAAACGAC	Used to increase the length of the homology region with *lacZ* for recombineering if needed
**Strains construction : $P_{LtetO-1}$-driven *mScarlet* fusions**		
Ptetno-10-cat-for	CACATGACGCGCTAGCTCCCTATCAGTGATAGAGATTGACATCCCTA TCAGTGATAGAGGCCAGGAATAGCCAGGAATTAAAATGAGACGTTG	Amplification of the [TT1-$P_{LtetO-1}$(no -10)-*cat-sacB*-TT2] cassette for construction of the MG2346 strain Amplification of *nptII*-FRT cassette for construction of the MG2348 strain
Ter-catsacRev	GATGTAAGGTTGAAAAATAAAAACGGCGCTAAAAAGCGCCGTTTTT TTTGACGGTGGTAATCAAAGGGAAAACTGTCCATATG	
Ter-Ptet-for	GGTACCAAATTCCAGAAAAGAGGCCTCCCGAAAGGGGGGCCTTTTT TCGTTTTGGTCCAACTAGTCCGATGCGACACACATGACGCGCTAGCT CCCTATC	
yhbX-Ter-rev	ATAAGCAAGGTAACCCCACCCCTGAAGGGCAGGGTTGATGTAAGGT TGAAAAATAAAAACGGCGCTAAA	
argG-Ter-for	GCCGCAGGTGGAGAATCTGGAAAACAAAGGCCAGTAATTCGCCTCG GTACCAAATTCCAGAAAAGAGGCCTCCCGAAAG	
sacB-KanR-For	CCTTGAACAAGGACAATTAACAGTTAACAAATAAAAACGCGAGGAT CGTTTCGCATGATTGAACAAG	Amplification of *nptII*-FRT cassette for construction of the MG2348 strain
FRT-sacB-Rev	CAAAGGGAAAACTGTCCATATGCACAGATGAAAACGGTGTAGTATG AATATCCTCCTTAGTTCCTATTCC	
Ptetno-10-mSC-For	GAGATTGACATCCCTATCAGTGATAGAGGCCAGGAATACATTCGGG CGTGAGCAAAGGCGAAG	Amplification of 'mScarlet-FRT cassette (with primer Ptet-55-12For) for construction of the MG2352 strain
mSc-FRT-Rev	GCGAAACGATCCTCATCCTGTCGAAGTTCCTATTCTCTAGAAAGTA TAGGAACTTCATTATTTATACAGTTCGTCCATGCC	
postFRT-KanR-Rev	GAACCTGCGTGCAATCCATCTTGTTCAATCATGCGAAACGATCCTCA TCCTGTC	
AK411	ATCAGTGATAGAGATTGACATCCCTATCAGTGATAGAGGCATTTGA GAAGCACACGG	*cat-sacB* amplification for construction of OK509 strain
AK412	ATTCTTTTATTACTGCTTCGCCTTTGCTCACGCCCGAATGAGACGTT GATCGGCAC	
AK420	AACGCATAAATTCTTTTATTACTGCTTCGCCTTTGCTCAC	$P_{LtetO-1}$-*btuB*$_{-240+210}$-*mScarlet*$_{+4}$ construction (with primers 5'PtetBtuB-240 and Ptet-55-12For)
AK451	CTGCTTCGCCTTTGCTCACCGGAAGACGGCGCAGCACATC	
5'PtetompR-35+30-lacZ	TAGAGATTGACATCCCTATCAGTGATAGAGATACTGAGCACTTACA AATTGTTGCGAACCTTTGGGAGT	$P_{LtetO-1}$*ompR*$_{-35+717}$-*mScarlet* $_{+4}$ construction
ompR+717-mScrev	GAAACGCATAAATTCTTTTATTACTGCTTCGCCTTTGCTCACTGCTT TAGAGCCGTCCGGTACAAAG	
Ptet-sdhC-222for	GACATCCCTATCAGTGATAGAGATACTGAGCACAGGTCTCCGGAAC ACCCTGCAATC	$P_{LtetO-1}$*sdhC*$_{-222+39}$-*mScarlet* $_{+4}$ construction
sdhC+39-mScrev	GAAACGCATAAATTCTTTTATTACTGCTTCGCCTTTGCTCACCAGAT TAACAGGTCTTTGTTTTTTCAC	
**Strains construction : *omrA/B* deletions**		
OmrA-kan3	CGCGAGCGACAGTAAATTAGGTGCGAAAAAAAACCTGCGCATCCGC GCAGGTTAGAAAAACTCATCGAGCA	a.k.a. ΔomrABrev in (10); *ΔomrA::nptI* construction
OmrA-kan5	CCCTTCATTCCTTTGCGTTTTCTCGCTGGCGAAGAGTCGTCGTGCAG ACAAAGCCACGTTGTGTCTCAA	*ΔomrA::nptI* construction
OmrB-kan3	CGCGAGCGACAGTAAATTAGGTGCGAAAAAAAACCTGCGCATCCGC GCAGGTTAGAAAAACTCATCGAGCA	*ΔomrB::nptI* construction
OmrB-kan5	GCGAAACGCTGTTGCGATTGACCGCTGGTGGCGTTTGGCTTCAGGT TGCAAAGCCACGTTGTGTCTCAA	a.k.a. ΔomrABfor in (10); *ΔomrB::nptI* construction
**Verification/sequencing**		
AK87	CCTTCTCCTGCTCTCCCTTAAGCG	*argG* check
AK88	CTCACGGGTTGTGGATGCAAAC	*argG* check
AK387	CTTCACCTTCACCCTCGATTTC	*mScarlet* fusions check

AK418	ACTGGCCTGCTTTCCTCCTC	*mScarlet* fusions check
AK430	CGTTCAGGAACGGATCTGC	*hfqQ8A* verification
AK431	TGGAACACGTTCCCGAGC	*hfqR16A* verification
AK432	GCTTAATACCATTCACCAAATC	*hfqY25D* verification
antiHfqout	GATGTGTACCAGTACCGCCT	*hfq* check (E. Hajnsdorf)
lacZ96-120(-)	GCTATTACGCCAGCTGGCGAAAGGG	*lac* fusions check
mHfqout	CAGGCGCGTGACGAAGTATT	*hfq* check (E. Hajnsdorf)
mhpR848-872	ATCTTCCGGCGCTACAACGGGTAGC	*lac* fusions check
seqOmrArev	GCATTCCAACTCCCTTTGCTC	*omrAB* check
seqOmrBfor	GGTGGCGTGTTTTCATCGTGG	*omrAB* check
**Plasmids construction**		
OmrAmut8for	GATTGGTGAGATTATTCGGGCTGGTACCCTGTCTCTTGCACC	Constructing pOmrAopt
OmrAmut8rev	GGTGCAAGAGACAGGGTACCAGCCCGAATAATCTCACCAATC	
OmrAmut9for	GGTATTGATTGGTGAGATTGCGAAATACCGTCTTCGTACCCTGTCTCTTG	Constructing pOmrAM9*
OmrAmut9rev	CAAGAGACAGGGTACGAAGACGGTATTTCGCAATCTCACCAATCAATACC	
OmrAmut8+9for	GATTGGTGAGATTGCGAAACGTGGTACCCTGTCTCTTGCACC	Constructing pOmrAoptM9*
OmrAmut8+9rev	GGTGCAAGAGACAGGGTACCACGTTTCGCAATCTCACCAATC	
OmrAmut11for	CCCAGAGGTATTGATTGGTCTCATTATTCGGTACGCTCTTCGTACCC	Constructing pOmrAoptM11*
OmrAmut11rev	GAAGAGCGTACCGAATAATGAGACCAATCAATACCTCTGGGGACGTC	
AK74	GAGGAGAAATTACATATGGCACGTTATTTCC	Constructing pCAS18n
AK75	GGAAATAACGTGCCATATGTAATTTCTCCTC	Constructing pCAS18n
**Templates for T7 *in vitro* transcription and toeprint**		
T7btuBfor	GGTAATACGACTCACTATAGCGCCAACGTCGCATCTGGTTCTC	For *in vitro* transcription of *btuB* (-240+61) region
btuBrev	ATATCCTGACGGGTCACAACGG	
btuBpT7 Fw	AATAATATCGACTCACTATAGGGGCCGGTCCTGTGAGTTAATAGG	For *in vitro* transcription of *btuB* (-240+61) region Mutant H1
btuB 301 Rev	GTGCCCAAGCGGAAAATGCC	
btuB 272 Rev	CACGCCGTCAGCAGCGAAGCGGTGGTGGTCATTGTAAAGCATCCAC	
btuB253.301 Rev	GTGCCCAAGCGGAAAATGCCGTGACGGAACACGCCGTCAGCAGCGAAGC	
T7OmrAfor	TAATACGACTCACTATAGGGCCCAGAGGTATTGATTGGTGAG	For *in vitro* transcription of *omrA*
OmrArev	AAAAAAAACCTGCGCATCCGCGC	
3995JG	GTGCCCAAGCGGAAAATGCC	Fig. 5E
**Templates for T7 *in vitro* transcription of radiolabeled probes for Northern blots (used in Fig. 1)**		
T7btuBprobe-for	TAATACGACTCACTATAGCGTACGCCATCAATTAACACCAAC	*in vitro* transcription of the antisense of *btuB* (+85+312) mRNA
btuBprobe-rev	GTCGTTACTGCTAACCGTTTTGAACAG	
T7OmrAprobe-for	TAATACGACTCACTATAGGGAAAAAAAACCTGCGCATCCGCGC	*in vitro* transcription of the antisense of OmrA sRNA
OmrAprobe-rev	CCCAGAGGTATTGATTGGTGAG	
T7OmrBprobe-for	TAATACGACTCACTATAGGGAAAAAAAACCTGCGCATCTGCGC	*in vitro* transcription of the antisense of OmrB sRNA
OmrBprobe-rev	CCCAGAGGTATTGATAGGTGAAGTC	
EM192	TAATACGACTCACTATAGGGAGATGCCTGGCAGTTCCCTACTC	*in vitro* transcription of the antisense of 5S rRNA
EM193	TGCCTGGCGGCAGTAGCG	
EM293	TAATACGACTCACTATAGGGAGACGCTTTACGCCCAGTAATTCC	*in vitro* transcription of the antisense of a 16S rRNA region
EM294	CTCCTACGGGAGGCAGCAGT	
**Biotinylated probes for Northern blots**		
3'OmrA-probe	BioTEG-CTGCGCATCCGCGCAGGTTGGTGCAAGAGACAGGGTAC	Fig. 4D, S6D
OmrA-probe	BioTEG-CAGGTTGGTGCAAGAGACAGGGTACGAAGAGCGTACCG	Fig. 4F, 5E, S5C and S14
OmrB-probe	BioTEG-CGCAGGCTGGTGTAATTCATGTGCTCAACCCGAAGTTGA	Fig. 4F, 5E and S14
Spot42-probe	BioTEG-GTAAAAGGTCTGAAAGATAGAACATCTTACCTCTGTACCC	Fig. 5F
SsrA-probe	BioTEG-CGCCACTAACAAACTAGCCTGATTAAGTTTTAACGCTTCA	Fig. 4F, 5F, S6D
5S-probe	BioTEG-CTACCATCGGCGCTACGGCGTTTCACTTCTGAGTTCG	Fig. 5E, S5C
